# Genome-wide identification, characterisation and expression profiles of calcium-dependent protein kinase genes in barley (*Hordeum vulgare* L.)

**DOI:** 10.1007/s13353-016-0357-2

**Published:** 2016-07-22

**Authors:** Olga Fedorowicz-Strońska, Grzegorz Koczyk, Małgorzata Kaczmarek, Paweł Krajewski, Jan Sadowski

**Affiliations:** 1 Institute of Plant Genetics, Polish Academy of Sciences, Strzeszynska 34, 60-479 Poznan, Poland; 2 Institute of Molecular Biology and Biotechnology, Adam Mickiewicz University, Umultowska 89, 61-614 Poznan, Poland

**Keywords:** Calcium-dependent protein kinases (CDPKs), Drought stress, Gene expression, *Hordeum vulgare*, Phylogenetic analysis

## Abstract

In plant cells, calcium-dependent protein kinases (CDPKs) are important sensors of Ca^2+^ flux resulting from various environmental stresses like cold, drought or salt stress. Previous genome sequence analysis and comparative studies in Arabidopsis (*Arabidopsis thaliana* L.) and rice (*Oryza sativa* L.) defined a multi-gene family of CDPKs. Here, we identified and characterised the CDPK gene complement of the model plant, barley (*Hordeum vulgare* L.). Comparative analysis encompassed phylogeny reconstruction based on newly available barley genome sequence, as well as established model genomes (e.g. *O. sativa*, *A. thaliana*, *Brachypodium distachyon*). Functional gene copies possessed characteristic CDPK domain architecture, including a serine/threonine kinase domain and four regulatory EF-hand motifs. In silico verification was followed by measurements of transcript abundance via real-time polymerase chain reaction (PCR). The relative expression of CDPK genes was determined in the vegetative growth stage under intensifying drought stress conditions. The majority of barley CDPK genes showed distinct changes in patterns of expression during exposure to stress. Our study constitutes evidence for involvement of the barley CDPK gene complement in signal transduction pathways relating to adaptation to drought. Our bioinformatics and transcriptomic analyses will provide an important foundation for further functional dissection of the barley CDPK gene family.

**Electronic supplementary material:**

The online version of this article (doi:10.1007/s13353-016-0357-2) contains supplementary material, which is available to authorized users.

## Introduction

One of the major features common to all organisms is the use of signal transduction pathways to control their metabolism and adapt to the changing environment. Frequently, calcium ions serve as a universal second messenger in such signal transduction pathways. The concentration of free, cytosolic Ca^2+^ in plant cells fluctuates in response to different stimuli, including hormones, pathogens, light and abiotic stresses (Evans et al. 2001; Sanders et al. 2002). These and other signals induce spatial and temporal Ca^2+^ spikes, as well as changes in the frequency and amplitude of Ca^2+^ oscillations.

Stimulus-specific increases in free, cytosolic Ca^2+^ levels are called ‘calcium signatures’ (Evans et al. 2001; Bose et al. 2011). Different Ca^2+^-interacting proteins recognise diverse calcium signatures and induce cascading downstream effects, such as altered protein phosphorylation and gene expression patterns. One of the largest and most differentiated group of calcium sensors are protein kinases, among them calcium-dependent protein kinases (CDPKs), which have been identified only in plants and protists (Harmon et al. 2000).

All members of this large multi-gene family have a conserved gene structure that consists of four characteristic conserved domains: the N-terminal domain, the serine/threonine kinase domain, the autoinhibitory domain and the calmodulin-like domain (Hrabak et al. 2003). The N-terminal domain is highly variable and often contains myristoylation or palmitoylation sites associated with subcellular targeting (Cheng et al. 2002). The conserved kinase domain is typical of serine/threonine kinases; its activation loop (located between subdomains VII and VIII) contains acidic residues, obviating the need for loop phosphorylation for kinase activity. The autoinhibitory domain contains a pseudosubstrate sequence capable of blocking the enzyme’s active site. The regulatory calmodulin-like domain contains four EF-hand motifs, each able to bind a single calcium cation (Klimecka and Muszyńska 2007). The CDPKs are often called sensor ‘responders’ as they are directly activated by calcium binding to the EF-hand motifs (Sanders et al. 2002). Subsequently, the conformation changes in the calmodulin-like domain lead to an induced conformational change in the kinase domain, which results in the displacement of the pseudosubstrate (autoinhibitory domain) from its active site (Reddy 2001).

The CDPKs are typically involved in the regulation of plant responses to a wide variety of stimuli, including hormones, cold/drought/salt stress, light and elicitor (Romeis et al. 2001; Lecourieux et al. 2006). A transgenic line of rice constitutively expressing *OsCDPK7* and *OsCDPK13* has enhanced tolerance to cold, salt and drought stress (Saijo et al. 2000; Komatsu et al. 2007), while *OsCPK23* (*SPK*) and *OsCPK19* (*OsCDPK2*) have been reported to be essential for seed development (Breviario et al. 1995; Frattini et al. 1999; Morello et al. 2000). In tobacco, CDPK-silenced plants show a reduced and delayed hypersensitive response to the fungal Avr9 elicitor (Romeis et al. 2001). Heterologous expression of a grape calcium-dependent kinase *ACPK1* in Arabidopsis provided evidence that the *ACPK1* gene is involved in abscisic acid (ABA) signal transduction as a positive regulator, and, thus, may be of use in improving plant biomass production (Yu et al. 2007). Two *Arabidopsis thaliana* guard cell-expressed CDPK genes, *AtCPK3* and *AtCPK6*, are involved in transduction of the stomatal ABA signal (Mori et al. 2006). Genetic evidence at the whole-plant level pointed to the *AtCPK4* and *AtCPK11* genes as positive regulators of the CDPK/calcium-mediated ABA signalling pathways (Zhu et al. 2007). The above evidence clearly indicates the role of the CDPK family in providing the basic building blocks for effective plant responses and increased plant resistance to abiotic and biotic stresses.

The Arabidopsis genome contains 34 genes encoding CDPKs (Cheng et al. 2002; Hrabak et al. 2003), while maize (*Zea mays* L.) contains 40 (Kong et al. 2013) and rice 29 (Asano et al. 2005) or 31 (Ray et al. 2007) CDPK genes. Such differences in the number of CDPK homologues may suggest different evolutionary processes (duplication, speciation, loss), leading to extant homologues with possibly divergent functional profiles in various species. To date, genome-wide identification of CDPK gene family members and analysis of their functional divergence has been carried out in only a few plants. Thus, our understanding of the evolution of the family of calcium-dependent proteins is still incomplete.

In this study, we identified the barley (*Hordeum vulgare*) complement of 27 CDPK genes and analysed their expression under water deficiency conditions. We revealed the significant differences in transcript levels of specific CDPK genes which implies their involvement in adaptation to drought stress. Multi-genome comparative analysis of the phylogeny and chromosomal distribution of CDPKs allowed us to propose evolutionary relationships (paralogy vs. orthology scenarios) and provide independent verification of gene correspondence and grouping of CDPKs from multiple reference plant genomes.

## Materials and methods

### Identification of CDPK genes in barley

The barley CDPK gene complement was annotated by using the BLASTn (Altschul et al. 1990) algorithm to search the Ensembl Plants database (genome assembly: 08214v1), as well as the FLcDNAs of the *H. vulgare* ‘Haruna Nijo’ cultivar expressed under normal and stressed conditions (Matsumoto et al. 2011) found in NCBI/GenBank (National Center for Biotechnology Information; http://www.ncbi.nlm.nih.gov). The candidate gene sequences were further identified via the IPK Gatersleben BLAST gateway (http://webblast.ipk-gatersleben.de/barley/viroblast.php), which includes a whole-genome assembly of *H. vulgare* cultivar ‘Morex’ (2,670,738 contigs; Mayer et al. 2011). CDPKs from other model organisms were annotated based on characteristic protein domain signatures, as revealed by searching the Ensembl Plant genomes against the Pfam v13 database (Punta et al. 2012) with HMMER 3.0 software (Finn et al. 2011). The precise exon/intron structures of CDPK genes were determined using Scipio (http://www.webscipio.org/) based on the corresponding protein sequences (Keller et al. 2008). The presence of myristoylation motifs at the N-terminal domain were predicted using the Eukaryotic Linear Motif resource (http://elm.eu.org; Dinkel et al. 2014). The localisations of ancestral duplications shared between barley and rice was determined by mapping previously identified sequences (Thiel et al. 2009) to the recently published genetically anchored physical map (International Barley Genome Sequencing Consortium 2012) (MEGABLAST vs. Ensembl Plants).

### Phylogeny reconstruction and reconciliation

**Fig. 1:**
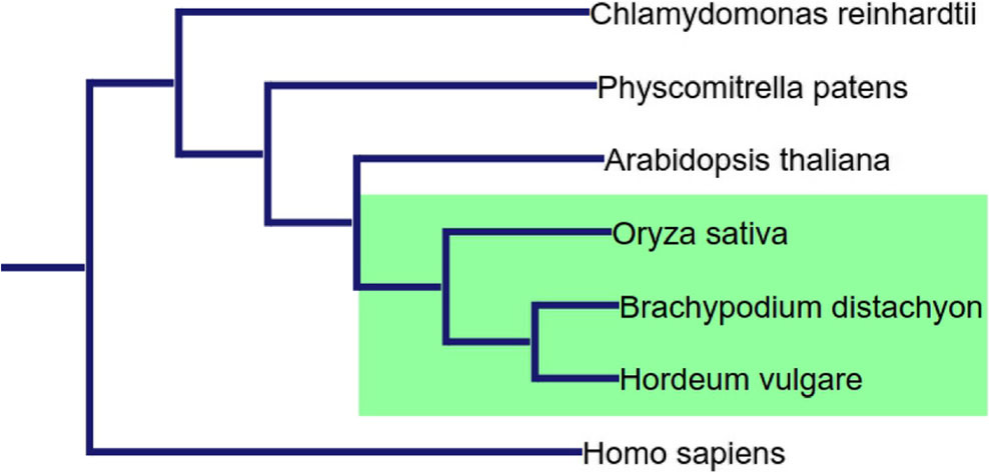
The reference species tree topology inferred from NCBI Taxonomy (approach analogous to Ensembl Compara; Sayers et al. 2009)

Comparative phylogenetic analysis was conducted on a set of six model plant genomes (*Chlamydomonas reinhardtii*, *Physcomitrella patens* ssp. *patens*, *Arabidopsis thaliana*, *Brachypodium distachyon*, *Oryza sativa* ssp. *japonica*, *Hordeum vulgare* ssp. *vulgare*). The multiple alignment of 143 CDPKs was prepared using the parallelised version of MAFFT-LINSI (Katoh and Toh 2010) and inspected in SeaView (Gouy et al. 2010). Conserved regions of the alignment were extracted with TrimAl (Capella-Gutiérrez et al. 2009) using a 70 % threshold for the exclusion of gapped sites (473 sites remained after exclusion). The resulting trimmed alignment is included in Online Resource 5. Maximum likelihood model parameters were assessed with ProtTest v3 (Darriba et al. 2011), according to both Akaike and Bayesian corrected information criteria. The final phylogenetic analysis was conducted in RAxML v.7.3 (Stamatakis 2006; Stamatakis et al. 2008), using the LG model of evolution (Le and Gascuel 2008), with fixed residue frequencies and a gamma-based site rates model. Tree support was computed based on 1000 bootstrap iterations (rapid bootstrap heuristic; Stamatakis et al. 2008). Human calmodulin-dependent kinase 1 was included as the outgroup (UniProt/SwissProt: KCC1A_HUMAN (Q14012)). The resulting gene tree was reconciled and visualised with a custom Python/ete2 script using a strict tree reconciliation algorithm (Page and Charleston 1997), as implemented in the ETE toolkit (Huerta-Cepas et al. 2010). The reference species tree topology was inferred from NCBI Taxonomy (approach analogous to Ensembl Compara; Sayers et al. 2009) and is depicted in Fig 1.

### Plant material and growth conditions

Experiments were carried out on two spring barley (*H. vulgare* L.) genotypes differing in response to drought stress: the drought-tolerant variety Sebastian and drought-susceptible variety Georgie (seeds kindly provided by Prof. Andrzej Górny, Institute of Plant Genetics collection). Sebastian, a variety of Danish origin, widely cultivated in the Czech Republic; and Georgie, a British variety released in 1975 (for details concerning varieties, see http://genbank.vurv.cz/barley/pedigree/). The seeds were surface-treated with JOCKEY 201FS for 5 min to protect them from fungal invasion and soaked in water for 24 h at 23 ± 2 °C with continuous shaking at 400 rpm to equalise germination. After treatment, the seeds were sown in 1-dm^3^ pots (ten plants each) filled with a mixture of sand and soil (2:7 w/w). The plants were grown in a greenhouse at 23/14 °C day/night, 55 % room humidity (RH), with a photoperiod of 10 h. The pots were watered and weighed every day and optimal soil moisture (8–12 %), corresponding to a soil moisture retention (pF) between 2.4 and 3.0, was maintained. The soil moisture retention curve (pF curve) was drawn for soil used in all experiments (kindly provided by Prof. Grzegorz Józefaciuk, The Bohdan Dobrzanski Institute of Agrophysics of Polish Academy of Sciences, Lublin, Poland) (Online Resource 1). It served to distinguish three stages of drought: mild at 3.2 pF, moderate at 3.6 pF and severe (beyond permanent wilting point) at 4.2 pF. Three-week-old plants were exposed to drought stress by withstanding of water to ensure a water content corresponding to 3.2 (first day of drought treatment), 3.6 (second day of drought treatment) or 4.2 pF (fourth day of drought treatment). The drought stress treatment was conducted in triplicate, where the pot was considered as a biological replicate. Each parameter combination (genotype * drought vs. control * stage of drought) was represented by ten plants per pot tested at the same time.

### Expression analysis

Samples of barley third leaves from ten plants were ground into fine powder in liquid N_2_ and total RNA was extracted using TRIZOL reagent according to the manufacturer’s manual (Life Technologies). The isolated RNA was purified with the SV Total RNA Isolation System (Promega). cDNA synthesis was performed in duplicate using a SuperScript cDNA Synthesis Kit (Invitrogen). The primers for the CDPK genes were designed using Primer3Plus software targeting the extreme 5′ end (extreme 5′ ends are not conserved), which produced an amplicon of 89–245 bp (primer length between 20 and 24 bp), with a melting temperature of 54–58 °C. Gene-specific primers used for each CDPK are shown in Online Resource 2. The sequence correctness of particular amplicons was verified by automatic sequencing. Quantitative reverse transcription polymerase chain reaction (RT-PCR) analysis was performed using the Stratagene Mx3000P Cycler system with Brilliant III Ultra-Fast SYBR QPCR MM Supermix (Stratagene) in a total volume of 20 μl. The reactions were performed as technical duplicates using independent cDNA synthesis reactions. Expression values were normalised against the ADP-ribosylation factor gene, which according to geNorm^PLUS^ analysis (data not shown), displayed the highest stability of expression level. It was also suggested as the most suitable to study drought-induced changes in gene expression at the seedling stage in barley (Rapacz et al. 2012). Expression values were calculated using the 2^−ΔΔCT^ method (Schmittgen and Livak 2008).

### Statistical analysis

For each gene independently, analysis of variance (ANOVA) of expression data was performed using the model of repeated measurements (Winer 1962) with main effects of variety (V), time of observations (T), drought treatment (D), and first- and second-order interactions. Significant effects were declared at *P* < 0.01. Drought effects (Drought – Control) were computed for all VxT combinations and used for the hierarchical grouping of CDPK genes visualised by a dendrogram (Euclidean distance, UPGMA algorithm). Computations were done in Genstat 15 (VSN International 2012).

## Results

### Barley CDPK genes: identification and chromosome distribution

**Fig. 2:**
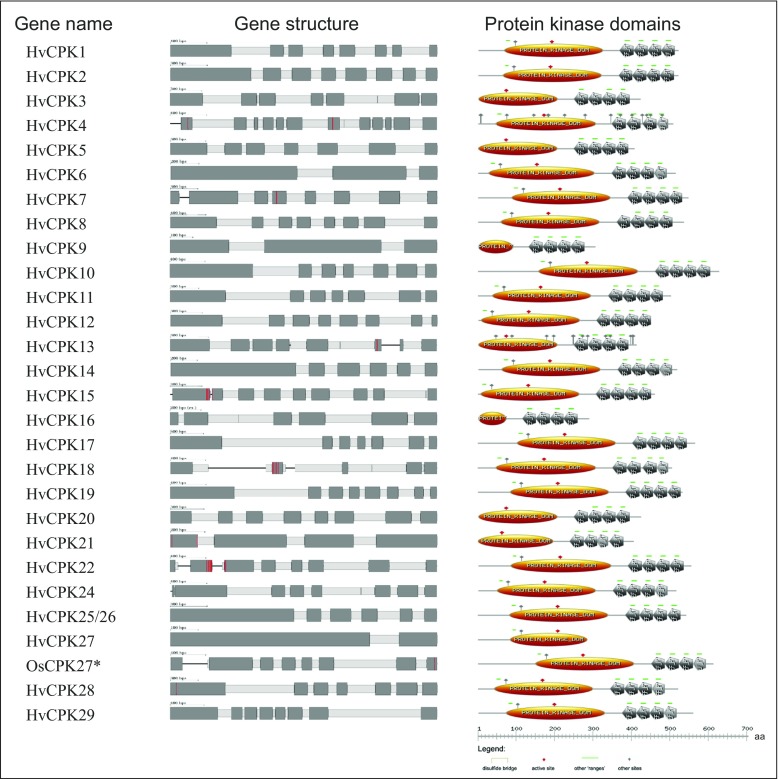
Genomic structures of barley CDPK genes. The intron/exon structures of CDPK genes was determined using Scipio (http://www.webscipio.org/), based on the protein sequence. Exons are marked with dark grey boxes. The ped lines indicate places where the coding sequence was not identified. The characteristic domains were visualised by PROSITE (http://prosite.expasy.org/; Sigrist et al. 2013). In case of incomplete sequence of *HvCPK27*, the structures of homologous *OsCPK27* were presented additionally

We conducted a genome-wide analysis of the barley CDPK gene family using the recently completed *H. vulgare* genome sequence (Mayer et al. 2011). Structural verification of the candidate CDPK protein sequences revealed a total of 27 genes (Table 1). The majority of them (19 genes with MLOC numbers) were annotated as protein kinases in the Ensembl Plants reference set (also supported by BarleyDB transcripts). However, three sequences were found only as low confidence gene candidates in the IPK Gattersleben dataset (supported by BarleyDB transcripts), while the remaining five candidates were found only among the BarleyDB transcripts available through the NCBI/GenBank database. Ten of the calcium-dependent kinase genes identified in the barley genome were found to contain myristoylation sites at their N-terminus (Table 1). Most of the barley CDPKs consisted of seven or eight exons, a pattern that is common to most plant CDPK genes (Fig. 2). Our proposed nomenclature of the newly annotated genes is based on similarity to corresponding rice kinases.

**Table 1:** Characteristics of CDPKs from barley

Name	Accession no.^a,b^	Chromosome^b^	Localisation^b^	cDNA length^a,b^	Protein ID^a,b^	Amino acids^a,b^	Mol wt (kDa)^a,b^	Myristoylation motif^c^
HvCPK1	MLOC_12765	3	409,133,979-409,137,786	1929	MLOC_12765.1	520	58.6	Yes
HvCPK2	MLOC_54510	3	465,728,019-465,732,215	2383	MLOC_54510.1	521	57.5	Yes
HvCPK3	MLOC_43400	3	499,953,131-499,962,423	1908	MLOC_43400.1	423	47.9	NI
HvCPK4	NIASHv1012I21/MLOC_72357	6	30,208,564-30,211,574	1432	BAK04063	376	42.4	Yes
HvCPK5	MLOC_68114	6	404,824,341-404,830,915	1992	BAJ96062	547	60.4	NI
HvCPK6	MLOC_6934	6HL	461,783-464,995	1952	MLOC_6934.1	514	56.5	NI
HvCPK7	AK249361	NA	NA	NA	NA	NA	NA	Yes
HvCPK8	MLOC_10811	5	528,571,776-528,578,065	2502	MLOC_10811.1	535	60	Yes
HvCPK9	MLOC_71733*	NA	NA	NA	NA	NA	NA	NI
HvCPK10	MLOC_55774	5	512,122,418-512,140,884	3705	MLOC_55774.3	627	68.2	NI
HvCPK11	MLOC_6391	5	512,512,790-512,521,346	2361	MLOC_6391.1	502	55.7	NI
HvCPK12	MLOC_76047	2	557,713,150-557,717,044	1560	MLOC_76047.2	448	50.4	NI
HvCPK13	MLOC_71042	2	580,049,260-580,054,582	1540	MLOC_71042.2	412	46	NI
HvCPK14	MLOC_76003	1	383,321,760-383,325,221	2012	MLOC_76003.1	517	57.3	NI
HvCPK15	MLOC_32468	1H	5,248,980-5,253,643	1607	MLOC_32468.	460	52.1	NI
HvCPK16	NIASHv1004P07	NA	NA	1407	BAJ85309	289	NA	NI
HvCPK17	MLOC_77271	2	481,950,289-481,957,075	2460	MLOC_77271.1	565	62.1	Yes
HvCPK18	NIASHv1023K05	NA	NA	1789	BAJ86849	506	NA	Yes
HvCPK19	MLOC_79572	2	495,288,610-495,295,072	2251	MLOC_79572.1	532	59.3	Yes
HvCPK20	MLOC_37356	2	246,284,388-246,289,165	1668	MLOC_37356.1	424	48.1	NI
HvCPK21	MLOC_7568	7	320,148,512-320,150,895	1366	MLOC_7568.1	389	44	NI
HvCPK22	NIASHv3144K21	NA	NA	2214	BAK08155	554	NA	Yes
HvCPK24	MLOC_38029*	NA	NA	NA	NA	NA	NA	NI
HvCPK25/26	MLOC_72770	5	67,817,230-67,820,564	1825	MLOC_72770.1	542	59.7	Yes
HvCPK27	MLOC_58648*	NA	NA	NA	NA	NA	NA	NI
HvCPK28	MLOC_59921	5	120,075,520-120,081,858	2449	MLOC_59921.1	520	57.3	NI
HvCPK29	MLOC_21560	5	263,659,893-263,668,678	2107	MLOC_21560.1	559	62.7	NI

*NI* not identified; *NA* data not available

^a^Barley DB at the National Center for Biotechnology Information (NCBI; http://www.ncbi.nlm.nih.gov)

^b^ *Hordeum vulgare* at Ensembl Plants (http://plants.ensembl.org/Hordeum_vulgare)

^c^Identified in the Eukaryotic Linear Motif resource (http://elm.eu.org)

*Sequence defined as low confidence gene

**Fig. 3:**
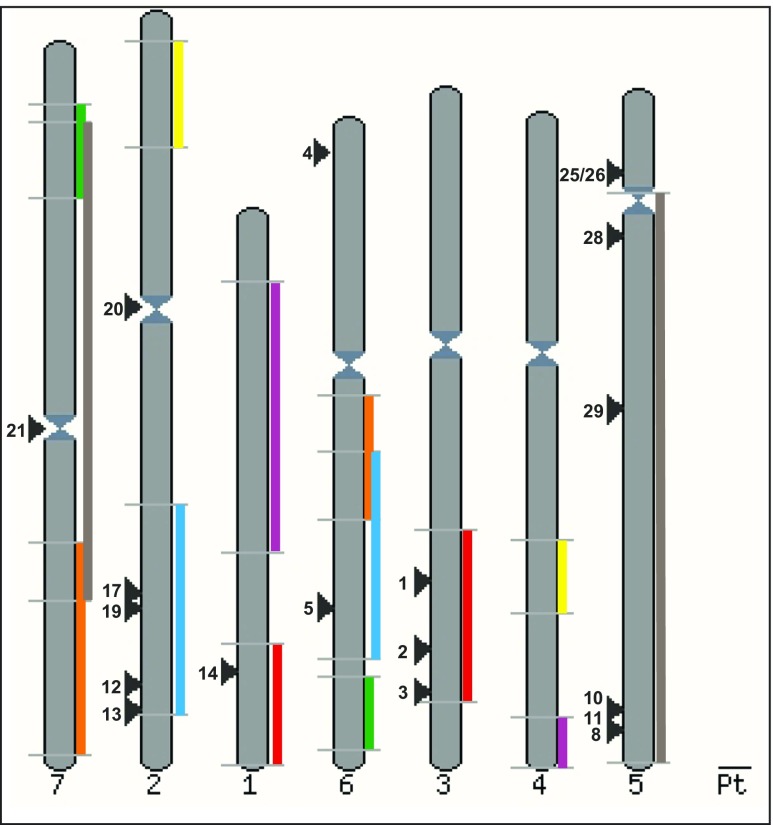
Chromosomal distribution of CDPK genes in the barley genome. The chromosome numbers are shown at the bottom. The black arrows and numbers indicate the approximate position of particular genes (MEGABLAST vs. Ensembl Plants). Ancestral duplicated genome segments determined on the basis of shared synteny between barley and rice (Thiel et al. 2009) are indicated by coloured boxes

**Fig. 4:**
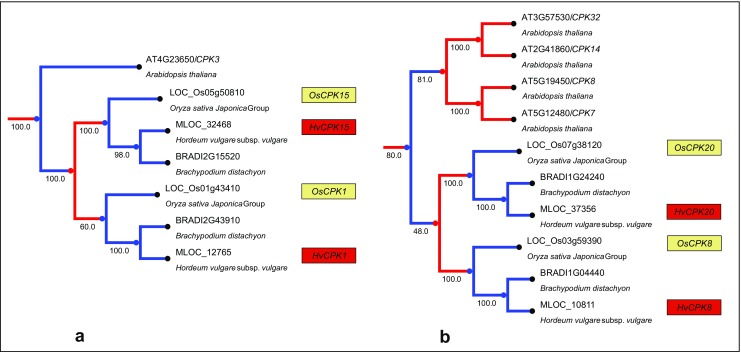
Orthologous relationships between monocot CDPKs. Rice and barley CDPK genes are indicated by numbers in yellow and red boxes, respectively, placed next to a particular accession number. The tree was rescaled for ultrametricity using the ETE toolkit. **a** Evolutionary relatedness of two orthologous monocot genes and Arabidopsis paralogue. **b** Evolutionary relatedness of two orthologous monocot genes and four Arabidopsis paralogues, which arose as a result of post-divergence duplications

The 18 high confidence genes were distributed among all barley chromosomes, save for chromosome 4 (Fig. 3). As many as 11 CDPK genes were found on just two chromosomes (2 and 5). Three additional genes were localised to chromosome 3, while two candidates were found on chromosome 6. The first and seventh chromosomes carry only one CDPK gene. No adjacent clusters of CDPK genes were observed, suggesting that these genes have not undergone tandem duplications in recent evolution involving genes encoding CDPKs. Notably, 15 out of 18 CDPK genes were localised within ancestrally duplicated genome segments (shared between barley and rice), suggesting that these kinase genes may have arisen via segmental duplication events predating the divergence of barley and rice. Three CDPK genes, namely *HvCPK20*, *HvCPK4* and *HvCPK25*/*26*, were not found on any of the ancestral duplicated genome segments, implicating either different scenarios (segmental duplication in rice only for *HvCPK25*/*26*, possible rearrangements for *HvCPK4* and *HvCPK20*) or mistakes in the annotation of the barley genome assembly, which is still under construction. Similarly, the close chromosomal positions of *HvCPK10* and *HvCPK11* (similar placement across all three monocots, divergence predating separation of monocot and dicot lineages; see Fig. 4 and Online Resource 5) suggests either a rearrangement or a segmental duplication predating said separation (in addition to monocot-specific duplications of individual genes).

### Phylogenetic analysis

Comparative analysis was conducted on a set of six plant genomes, which enabled study of the evolutionary relationships between the recently annotated barley CDPK complement and the reannotated sets of CDPK genes from well-characterised model species (e.g. *O. sativa* and *A. thaliana*) (Online Resources 3–5). The final analysis was based on conserved regions of the corresponding protein sequence (>70 % representation in multiple alignment) (Online Resource 6). The support at most key nodes of the resolved tree was high; in particular, CDPK groups I–VI were all well-supported clades. The first four monophyletic groups have been reported previously (Asano et al. 2005; Li et al. 2008), while groups V (unique to *C. reinhardtii*) and VI (unique to *P. patens*) are novel. However, the ancient events corresponding to divergence between ancestral CDPK groups are not satisfactorily resolved by our analysis (support for the respective bipartitions is below 50 %). Since the mapping step of reconciliation (Page 1994) is a top-to-bottom approach where later events are resolved first, low confidence in the order of these early bipartitions does not impact the resolution of individual duplication/speciation events closer to the leaves.

Notably, the analysis points to independent duplications and subsequent diversification of CDPK complements in all major lineages analysed (algae, mosses, dicots, monocots) (Online Resources 3–5). The *C. reinhardtii* genome has its own monophyletic, extensively duplicated CDPK complement (group V). In the case of *P. patens*, the extensively duplicated monophyletic group VI, as well as subclades within groups I and III, are unique to this model moss genome. The dicot CDPKs (inferred from the *A. thaliana* genome) are typically paralogous (with multiple copies retained) relative to monocots. Subgroups that were previously distinguished in groups II and III (Li et al. 2008; Ray et al. 2007) are not validated by this reconstruction.

**Fig. 5:**
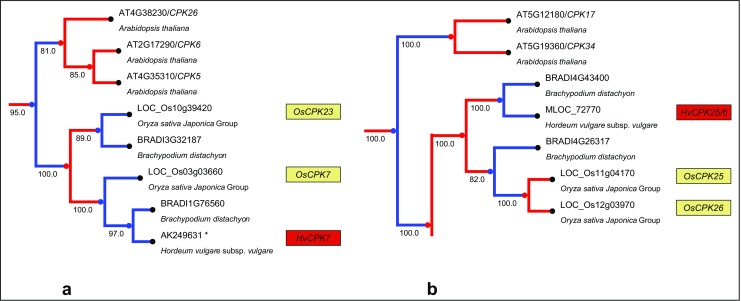
Paralogous relationships between monocot CDPKs. Rice and barley CDPK genes are indicated by numbers in the yellow and red boxes, respectively, placed next to a particular accession number. The tree was rescaled for ultrametricity using the ETE toolkit. **a** Clad containing *OsCPK23* and *OsCPK7* genes where absence of the orthologue of OsCPK23 in barley genome was identified. **b** Clad containing genes *OsCPK25*, *OsCPK26* and their paralogues, which arose as a result of complex duplication and gene loss processes

Phylogenetic analysis based on amino acid sequences distinguished closely related pairs of barley CDPKs: *HvCPK4*/*18*, *HvCPK1*/*15*, *HvCPK2*/*14*, *HvCPK3*/*16*, *HvCPK21*/*22*, *HvCPK8*/*20*, *HvCPK24*/*28*, *HvCPK11*/*17*, *HvCPK10*/*27* and *HvCPK5*/*13* (Online Resources 3–5). The majority of CDPKs in the monocots examined are orthologous (Fig. 4), but two exceptions were noticed (Fig. 5). First, an orthologue of *OsCPK23* was not found in barley. Second, the genes *OsCPK25* and *OsCPK26* represent an exception in that they are most likely the result of a recent duplication after the separation of the rice and barley lineages. Additionally, the presence of one gene in the barley genome (defined as *HvCPK25*/*26*) and two paralogues in *B. distachyon* indicates a complex gene duplication and gene loss scenario.

### Barley CDPK gene expression

**Fig. 6:**
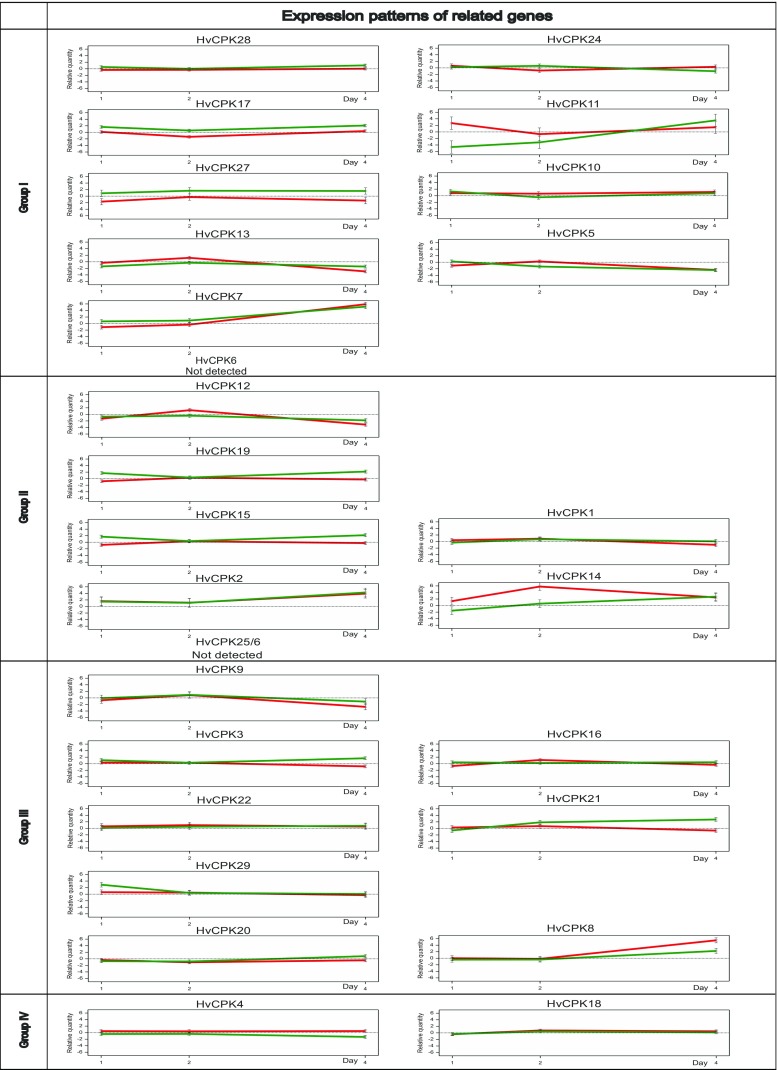
Barley CDPK gene expression profiles in response to intensifying drought stress conditions detected in Sebastian (green line) and Georgie (red line) varieties. Numbers of days (1, 2 and 4) correspond to a field capacity (pF) of 3.2, 3.6, and 4.2, respectively. Relative quantification was determined by Q-PCR analysis (with ADP-ribosylation factor gene as internal controls). Vertical bars correspond to standard errors of the mean values

We quantified transcript levels of the majority of the studied genes (25 CDPKs) by quantitative PCR (Q-PCR). However, in most cases, we observed low levels of expression and, indeed, there was no expression of *HvCPK6* and *HvCPK25*/*26* at all. In leaf tissues, the 25 CDPK genes responded differently to water deficit: detailed expression profiles over time are presented in Fig. 6. In particular, three genes (*HvCPK7*, *HvCPK8* and *HvCPK2*) were markedly upregulated. As expected, the shape of the expression profile (e.g. *HvCPK24*, *HvCPK19*) or the expression level (e.g. *HvCPK17*, *HvCPK27*) of different CDPK genes depended on the variety of barley. However, eight CDPK genes had similar expression patterns in the two varieties under study.

Analysis of variance (ANOVA) showed 19 significant main drought effects or their modifications by variety (Sebastian and Georgie) or time for 11 genes (Table 2). The ANOVA was carried out for subsets of time points and showed that seven effects (for seven genes) and 18 effects (for ten genes) could be declared significant when considering only days (1, 2) and (2, 4), respectively. This shows that significant changes in gene expression take place mainly during the later stages of drought.

**Table 2:** ANOVA results (*F*-test *P*-values) for observations of CDPK gene expression in two barley varieties under drought and control conditions

Gene symbol	Group	Source of variation			
		D	VD	TD	VTD
*HvCPK5*	I	<0.001	–	0.005	–
*HvCPK7*	I	<0.001	–	<0.001	–
*HvCPK10*	I	–	–	–	–
*HvCPK11*	I	–	–	–	–
*HvCPK13*	I	<0.001	–	0.001	–
*HvCPK17*	I	0.005	<0.001	–	–
*HvCPK24*	I	–	–	–	–
*HvCPK27*	I	–	–	–	–
*HvCPK28*	I	–	0.005	–	–
*HvCPK1*	II	–	–	–	–
*HvCPK2*	II	–	–	–	–
*HvCPK12*	II	<0.001	–	0.001	–
*HvCPK14*	II	<0.001	0.003	–	–
*HvCPK15*	II	–	–	–	–
*HvCPK19*	II	0.003	<0.001	–	–
*HvCPK3*	III	0.005	0.003	–	–
*HvCPK8*	III	–	–	<0.001	–
*HvCPK9*	III	–	–	–	–
*HvCPK16*	III	–	–	–	–
*HvCPK20*	III	0.003	–	–	–
*HvCPK21*	III	–	–	–	–
*HvCPK22*	III	–	–	–	–
*HvCPK29*	III	–	–	–	–
*HvCPK4*	IV	–	–	–	–
*HvCPK18*	IV	–	–	–	–

– Non-significant, *D* drought effect, *VD* drought effect modified by variety, *TD* drought effect modified by time point, *VTD* drought effect modified by variety and time point

The members of a pair of gene homologues are either expressed differently or similarly to each other, implying that either sub-functionalisation or conservation of expression patterns, respectively, has occurred after the duplication event. In the case of one pair (*HvCPK5*/*13*), this similarity of expression patterns was supported by ANOVA, showing that the effects of drought and drought modified by time point are significant for both gene homologues.

## Discussion

CDPK activity was first reported in shoot membranes of pea (*Pisum sativum*; Hetherington and Trewavas 1982) and has since been identified and characterised in many plants and some protozoa (Harmon et al. 2000). To date, the full set of CDPKs has not been described for barley, chiefly due to limited genome sequence information. The recent publication of an ordered, information-rich scaffold of the barley genome (Mayer et al. 2011) provided a valuable framework for the research described in this paper.

All identified sequences share a high degree of protein and nucleotide similarity and possess typical CDPK protein architecture, except an incomplete sequence of *HvCPK27* annotated on the basis of high sequence similarity to the rice gene (*OsCPK27*), which contains only the N-terminal and kinase domain. The majority of barley CDPKs (19) were identified in the Ensembl Plants *H. vulgare* reference set, while three further sequences were found in the low confidence gene subset (containing potential gene fragments). The five genes were corroborated solely by BarleyDB data (Matsumoto et al. 2011). These sequences could not, however, be directly associated with the genetically anchored physical map (International Barley Genome Sequencing Consortium 2012). In previous studies, such in silico analysis of transcriptome data enabled the identification of four *H. vulgare* EST contigs representing full-size CDPK family members expressed in the barley leaf epidermis (Freymark et al. 2007).

The CDPK-encoding genes with assigned genome locations (18) are, like in rice (Asano et al. 2005), randomly dispersed throughout the genome. This observation, together with the high sequence similarity of all barley CDPK genes, indicates that they were derived from segmental rather than tandem duplication. Conversely, in the case of *A. thaliana*, a set of five genes (*AtCPK31*, *AtCPK27*, *AtCPK22*, *AtCPK21* and *AtCPK23*) classified in the same monophyletic group is tandemly arranged in the same transcriptional orientation on chromosome 4, indicating that they may have arisen from a relatively recent gene duplication (Cheng et al. 2002).

The evolutionary relationships outlined in our study are consistent with independent diversification of CDPK complements in all major lineages analysed via extensive duplication(s) and differential loss of resulting copies. A likely origin of the observed copies lies in the ancestral whole-genome duplications inferred at the base of many extant lineages. Such an explanation is in line with the documented tendency for organisms to preferentially retain neofunctionalised duplicates of regulatory toolkit components (such as kinases) arising from whole-genome duplication events (Jiao et al. 2011, 2012; Tang et al. 2010). In particular, the resolution of triplets (close homologues present in all three model monocot genomes) indicates that the respective ancestral duplications most often took place before the divergence of monocot genomes (about 50–70 MYA; Kellogg 2001). Consequently, dicot CDPKs (as typified by *A. thaliana*) are typically paralogous to those of monocots. This result, together with the aforementioned differences in chromosome distribution, indicates that dicot CDPKs should not be employed as a model in studies based on orthology-derived assumptions. The present evidence also points to the majority of monocot CDPKs being orthologous, although there are some exceptions. For example, the absence of an *OsCPK23* orthologue seems to be barley-specific, as a counterpart of this gene is present in *B. distachyon*. Furthermore, the *OsCPK25* and *OsCPK26* sequences are very similar to each other at the nucleotide level (99.3 %) (Asano et al. 2005) and are located in the duplicated regions of chromosomes 11 and 12, respectively. Therefore, *OsCPK25* and *OsCPK26* may have arisen through a recent rice-specific segmental duplication. The two paralogues present in *B. distachyon* most likely arose before monocot divergence, implicating a complex scenario of duplication and independent loss in the different monocot lineages.

Based on sequence similarity, pairs of CDPK genes can be distinguished: ten in barley and 12 in both rice and *B. distachyon*. The ubiquity of duplicated CDPK genes in the studied monocot species indicates that these duplications took place before the barley–rice and barley–*B. distachyon* lineage separations. This also implies that the current complement evolved as a consequence of selection rather than random drift, because gene duplicates should have a short lifespan without selection (Force et al. 1999). The divergence time of rice CDPK gene pairs, estimated on the basis of the individual duplication events, is considered to be ∼50 MYA (Ramakrishna et al. 2002).

The results of our comparative phylogenetic analysis point to the majority of monocot CDPKs being orthologous and placed within ancestrally duplicated regions previously identified between barley and rice (Thiel et al. 2009). Including more species in subsequent studies will probably increase the resolution of duplication events, but it is unlikely to challenge the hypothesis of independent diversification of CDPK complements.

Our expression analysis indicates that almost all of the barley CDPK genes identified are functionally active (25 of 27 genes). The two genes for which transcripts were not detected (*HvCPK6* and *HvCPK25*/*26*) may be expressed in different plant tissues, or only in response to certain stimuli, or at a specific developmental stage. It has been reported (Ray et al. 2007) that rice homologues of these genes are expressed during panicle development. The evidence for *HvCPK25*/*26* being expressed specifically in flower organs is supported by the observation that full-length cDNA corresponding to this gene deposited in BarleyDB was, indeed, isolated from early stage flowers (Matsumoto et al. 2011). Such data are, unfortunately, not available for *HvCPK6*.

The number of barley CDPK genes responds differently to water deficiency: ANOVA indicates that, for 11 genes, the drought effect or its modifications by variety or time are significant. This suggests that multiple CDPKs are regulated in a coordinated response to a single stress stimulus. The expressed kinases localise in many different cellular compartments (Harper et al. 2004). Additionally, CDPKs also differ in their affinity for Ca^2+^ ions (Hrabak et al. 1996). Their sensitivity to calcium can be modulated both by the type of protein substrate and by defects in one or more of their EF hands. The observed differences in affinity might mean that each calcium-dependent protein responds to a specific set of calcium signals, which, in turn, differ in frequency of oscillation, magnitude and duration, depending on the stimulus (McAinsh and Pittman 2009). Thus, different CDPKs even within the same subgroup may have distinct roles at different stages of a plant’s reaction to biotic and abiotic stresses. Calcium-dependent proteins are involved in complex interactions with mitogen-activated protein kinase (MAPK) cascades and other sensors. For example, the different Ca^2+^ signatures associated with diverse microbe-associated molecular patterns (MAMPs) may be decoded by distinct CDPKs and, thus, partially account for differential MAMP responses (Boudsocq et al. 2010). Additionally, a given signal can induce a different Ca^2+^ signature in different cell types (Kiegle et al. 2000) and, consequently, affect a different response in downstream signalling pathway components. Moreover, highly modulated plant responses to environmental stimuli are likely the outcome of cross-talk between Ca^2+^-dependent and Ca^2+^-independent transduction pathways (Mehlmer et al. 2010). Taking into account the above, our observations confirm that regulatory CDPKs should be regarded as multi-functional genes that partake in complicated signalling networks affecting a specific response through different calcium sensitivities, expression, cellular localisations and substrate regulation.

In water deficiency conditions, the barley CDPK genes *HvCPK7*, *HvCPK8* and *HvCPK2* were highly induced, which implies their involvement in drought stress signalling and adaptation. Similar studies on rice (Ray et al. 2007; Wan et al. 2007) showed that the *OsCPK13* gene was always highly upregulated by drought stress. Additionally, overexpression of this gene was found to confer significant cold and drought/salt tolerance on rice plants (Saijo et al. 2000). In wheat (*Triticum aestivum* L.), the orthologue of *OsCPK13* (*TaCPK2*) seems to have lost its ability to respond to biotic stresses, while three other CDPK genes (*TaCPK1*, *TaCPK6* and *TaCPK9*; homologues of *OsCPK7*, *OsCPK18* and *OsCPK11*, respectively) were upregulated by drought (Li et al. 2008). Such discrepancies in expression levels between rice, wheat and barley CDPKs indicate that the regulatory competences of particular kinases are likely species-specific. Moreover, our analysis of two varieties of barley suggests that CDPK gene regulation can also be genotype-specific. Previously functional studies in barley have demonstrated that two CDPK paralogues, namely *HvCDPK3* and *HvCDPK4* (*HvCPK19* and *HvCPK13*, in this study, respectively), play antagonistic roles during the early phase of powdery mildew pathogenesis (Freymark et al. 2007). In addition, *HvCDPK1* (*HvCPK1*) has already been implicated in the gibberellic acid response of the barley aleurone through regulation of vacuolar function (McCubbin et al. 2004). In the context of our results, these findings indicate that the same calcium-dependent proteins can be involved in different signal transduction pathways, as well as being implicated in adaptations to differing stimuli.

In summary, the barley CDPK complement of 27 genes was identified and characterised in this study. The different pattern of response of the CDPK genes under water deficiency conditions constitutes evidence for their involvement in signal transduction pathways relating to adaptation to drought. A precise definition of the role of CDPK genes in transduction pathways requires further studies, including the definition of isoform-specific calcium activation thresholds, substrate specificities and subcellular locations.

## Electronic supplementary material

Below are the links to the electronic supplementary material.

ESM 1 The soil water retention curve (pF curve) drawn for soil used in the study (kindly provided by Prof. Grzegorz Józefaciuk, The Bohdan Dobrzanski Institute of Agrophysics of Polish Academy of Sciences, Lublin, Poland). The black ovals designate pF values at which plant material was taken. (JPG 23 kb)

ESM 2 Gene-specific primers used in real-time polymerase chain reaction (PCR) amplification. (PDF 269 kb)

ESM 3 Comparative phylogenetic tree constructed on a set of six model plant genomes (*Chlamydomonas reinhardtii*, *Physcomitrella patens* ssp. *patens*, *Arabidopsis thaliana*, *Brachypodium distachyon*, *Oryza sativa* ssp. *japonica*, *Hordeum vulgare* ssp. *vulgare*): **a**, **b** and **c**, respectively. (PDF 2180 kb)

ESM 4 Comparative phylogenetic tree constructed on a set of six model plant genomes (*Chlamydomonas reinhardtii*, *Physcomitrella patens* ssp. *patens*, *Arabidopsis thaliana*, *Brachypodium distachyon*, *Oryza sativa* ssp. *japonica*, *Hordeum vulgare* ssp. *vulgare*): **a**, **b** and **c**, respectively. (PDF 1904 kb)

ESM 5 Comparative phylogenetic tree constructed on a set of six model plant genomes (*Chlamydomonas reinhardtii*, *Physcomitrella patens* ssp. *patens*, *Arabidopsis thaliana*, *Brachypodium distachyon*, *Oryza sativa* ssp. *japonica*, *Hordeum vulgare* ssp. *vulgare*): **a**, **b** and **c**, respectively. (PDF 2077 kb)

ESM 6 The multiple alignments of analysed sequences. (PDF 112 kb)
