# Supplementary figures and images for: Genome-wide identification, characterisation and expression profiles of calcium-dependent protein kinase genes in barley (Hordeum vulgare L.)
Source: J Appl Genet. 2016 Jul 22;58(1):11–22. doi: 10.1007/s13353-016-0357-2 (PMC5243917; doi:10.1007/s13353-016-0357-2)

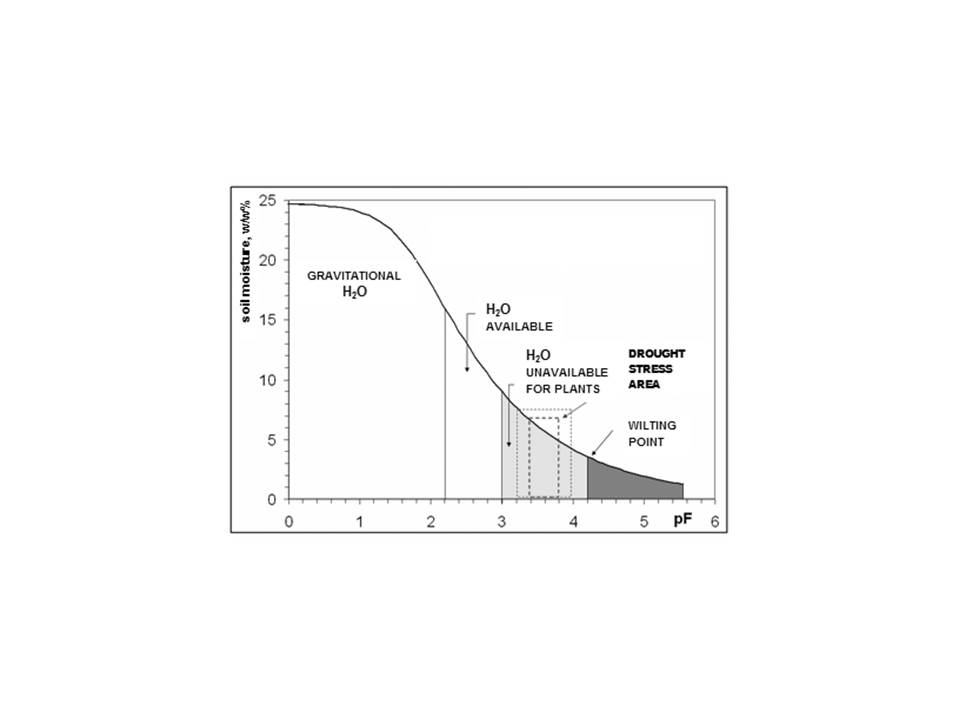

Supplement: Supplementary file 1 — The soil water retention curve (pF curve) drawn for soil used in the study (kindly provided by Prof. Grzegorz Józefaciuk, The Bohdan Dobrzanski Institute of Agrophysics of Polish Academy of Sciences, Lublin, Poland). The black ovals designate pF values at which plant material was taken. (JPG 23 kb) [file 13353_2016_357_MOESM1_ESM.jpg]
