# Supplementary material for: Genome-wide identification, characterisation and expression profiles of calcium-dependent protein kinase genes in barley (Hordeum vulgare L.)
Source: J Appl Genet. 2016 Jul 22;58(1):11–22. doi: 10.1007/s13353-016-0357-2 (PMC5243917; doi:10.1007/s13353-016-0357-2)
Supplement: Supplementary file 2 — Gene-specific primers used in real-time polymerase chain reaction (PCR) amplification. (PDF 269 kb) [file 13353_2016_357_MOESM2_ESM.pdf]

Genome-wide identification, characterization and expression profiles of calcium-dependent protein kinase genes in barley (*Hordeum vulgare L.*)

Olga Fedorowicz-Strońska, Grzegorz Koczyk, Małgorzata Kaczmarek, Paweł Krajewski, Jan Sadowski
Corresponding author: Olga Fedorowicz-Stronska, Institute of Plant Genetics, Polish Academy of Sciences, Strzeszynska 34, 60-479 Poznan,
Poland E-Mail: ofed@igr.poznan.pl, Tel.: +48-61-6550251; Fax: +48-61-6550301

Gene-specific primers used for QPCR amplification

Name of the gene	Forward primer sequence	Reverse primer sequence	Product length (bp)
HvCPK1	5'-CTTCCTGGCCCCATTATCTT-3'	5'-GATCAGGAGGCAGTACGACA-3'	161
HvCPK2	5'-GGGCAAGCTACACAAAGGAG-3'	5'-ACGTAGCCGTGGAGGTTGTA-3'	100
HvCPK3	5'-AAGGCATCACGGCATTATTC-3'	5'-CCACACAAAACGACCATGAG-3'	184
HvCPK4	5'-CACCCCCGATGAACTTAGAA-3'	5'-AGTGTACCCACACCGTCTCC-3'	227
HvCPK5	5'-TGGAGAGTTTGTTGCGATGA-3'	5'-ATGCCCACACGTACAACTGA-3'	153
HvCPK6	5'-GACGACATCATCAGGGAGGT-3	5'-AGAACGCTACCATCGGAAGT-3'	134
HvCPK7	5'-AAGATATGGCAACGGATTGG-3'	5'-GGAGAGTGCCCTCCATGTTA-3'	242
HvCPK8	5'-CTGGCCTTCGTAAACTTGGA-3'	5'-CATCGGTCAGTGCCACTCTA-3'	231
HvCPK9	5'-GACGGCTACCTCGACTATGC-3'	5'-ACGAACTCCTCGAAGCTGAC-3'	245
HvCPK10	5'-CTGAGGATGGCTTGAAGGAG-3'	5'-CCTGCCTGCCTCTAACACAG-3'	209
HvCPK11	5'-GCGAGGAGTTTGGTATTGGA-3'	5'-TTGCTTTGCTGTCCCTTTCT-3'	148
HvCPK12	5'-GGATGGGTGATGAAGCAAAT-3'	5'-CATCAAGAGGCCCAAGTTTC-3'	137
HvCPK13	5'-CATGATGACCAAAGGCAACA-3'	5'-CACACATCCACACAGGCATT-3'	202
HvCPK14	5'-CGTGGTAGGTAGGCACACAA-3'	5'-GGGGTAAACACGAGGAAACA-3'	234
HvCPK15	5'-ATGGGTGATGAGGCAACAAT-3'	5'-CAACGCATTCACAGAAATCG-3'	188
HvCPK16	5'-GGATGGCAAAATTAGCTTCG-3'	5'-TGTGCGATTTCACTCGTTTC-3'	165
HvCPK17	5'-CCTTACTATGTCGCCCCTGA-3'	5'-TCTTTCCAGGATCACGGTTC-3'	244
HvCPK18	5'-GGTCATGCAACCCAACTCCA-3'	5'-GAAGCCCCTCTGGCAACATTT-3'	139
HvCPK19	5'-ACATTTGTTCAAGGCGTTCC-3'	5'-TCCTCTCATCATAGCGCAGA-3'	190
HvCPK20	5'-GCCATCATCCGCGATGTAGA-3'	5'-TGCAACGATCCATCCTTCTGC-3'	164
HvCPK21	5'-AGGTGATCGACTTCGGACTG-3'	5'-ACCGCAGAGGAGGATGTAGA-3'	164
HvCPK22	5'-CTGGCTCAGATTGGAGGAAT-3'	5'-GCTTGGATCGGTCTTGTTTC-3'	107
HvCPK24	5'-TGGATGCCAGTTTGTGAAAG-3'	5'-ACAACCTGCAACCACATGAA-3'	207
HvCPK25/26	5'-AGCTGGAGCAAGCCTTACAA-3'	5'-TAGGTCTCGCCTCTTCTTCG-3'	176
HvCPK27	5'-CGTAAGACCGACAACCTCAAG-3'	5'-TTGTCCACGCACAGGTATGT-3'	89
HvCPK28	5'-GCATGCGAGAAATTTGGTCT-3'	5'-ACTGGAGCCACCAGCATTAC-3'	135
HvCPK29	5'-GCTCCAAACATTCCTCTTGG-3'	5'-ACAGTTCCGTGATTGCTTCC-3'	139
ADP-ribosylation	5'-TGCTGAATGAGGATGAGCTG-3'	5'-GTCCCTCGTACAACCCTTCA-3'	180
