# Supplementary material for: Genome-wide identification, characterisation and expression profiles of calcium-dependent protein kinase genes in barley (Hordeum vulgare L.)
Source: J Appl Genet. 2016 Jul 22;58(1):11–22. doi: 10.1007/s13353-016-0357-2 (PMC5243917; doi:10.1007/s13353-016-0357-2)
Supplement: Supplementary file 3 — Comparative phylogenetic tree constructed on a set of six model plant genomes (Chlamydomonas reinhardtii, Physcomitrella patens ssp. patens, Arabidopsis thaliana, Brachypodium distachyon, Oryza sativa ssp. japonica, Hordeum vulgare ssp. vulgare): a, b and c, respectively. (PDF 2180 kb) [file 13353_2016_357_MOESM3_ESM.pdf]

Genome-wide identification, characterization and expression profiles of calcium-dependent protein kinase genes in barley (*Hordeum vulgare L.*)

Olga Fedorowicz-Strońska, Grzegorz Koczyk, Małgorzata Kaczmarek, Paweł Krajewski, Jan Sadowski

Corresponding author: Olga Fedorowicz-Stronska, Institute of Plant Genetics, Polish Academy of Sciences, Strzeszynska 34, 60-479 Poznan, Poland E-Mail: ofed@igr.poznan.pl, Tel.: +48-61-6550251; Fax: +48-61-6550301

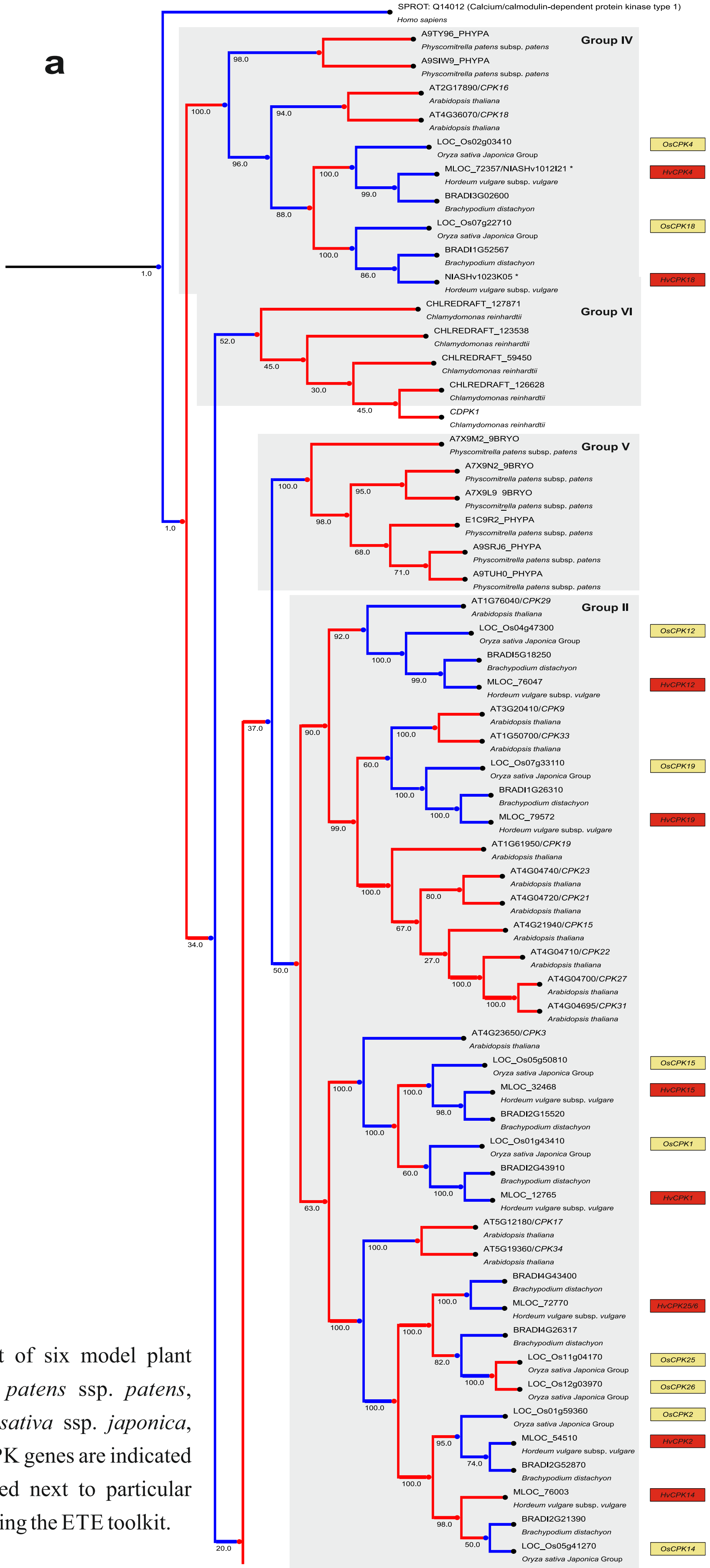

Comparative phylogenetic tree was constructed on a set of six model plant genomes (*Chlamydomonas reinhardtii*, *Physcomitrella patens* ssp. *patens*, *Arabidopsis thaliana*, *Brachypodium distachyon*, *Oryza sativa* ssp. *japonica*, *Hordeum vulgare* ssp. *vulgare*) - part a. Rice and barley CDPK genes are indicated by numbers in yellow and red boxes, respectively, placed next to particular accession number. The tree was rescaled for ultrametricity using the ETE toolkit.
