# Supplementary material for: Genome-wide identification, characterisation and expression profiles of calcium-dependent protein kinase genes in barley (Hordeum vulgare L.)
Source: J Appl Genet. 2016 Jul 22;58(1):11–22. doi: 10.1007/s13353-016-0357-2 (PMC5243917; doi:10.1007/s13353-016-0357-2)
Supplement: Supplementary file 6 — The multiple alignments of analysed sequences. (PDF 112 kb) [file 13353_2016_357_MOESM6_ESM.pdf]

```
>LOC_Os01g43410 -1001737
MGNRAPDAAMGRVLGRPMEDVRATYTFGRELGRGQFGVTYLVTHKATGKRFACKSIATRKLAHRDD[illegible]
>LOC_Os01g59360 -1003602
MGNCKPPAPIGPVLGRPMEDVRSIYTIGKELGRGQFGVTSLCTHKATGQKFACKTIAKRKLSTKED[illegible]
>LOC_Os01g61590 -1003858
MGNCGAGQKRLPVLGEE-GGIDDKYALDRELGRGEFGVTYLCMDRDTKELLACKSISKRKLRTAVD[illegible]
>MLOC_21560 -1008619
MGNCAQPWSSVTVLGKGIEELLRRYQLGEELGRGEFGVTRRCMDAATGEKLACKSISKRKLRSSVD[illegible]
>MLOC_6934 -1012234
MGRG----SPTAVLGHHTPPLRDLYSLGRKLGQGQFGTTYLCTETATGTAFACKSIAKRKLLTPED[illegible]
>MLOC_76047 -1017508
----------------MADVRALYNLERKLGSGQFGTTYLCTERATGLKYACKSVSKRKLVRRAD[illegible]
>MLOC_32468 -1023933
----------------MEDVRATYTFGRELGRGQFGVTYLATHKSTGARYACKSIAARKLARADD[illegible]
>MLOC_76003 -1028083
MGMCKPPAPIGPVLGRPMEDVKSIYNVGKELGRGQFGVTSLCTQKATGQKLACKTISKRKLSTKED[illegible]
>MLOC_68114 -1033069
[illegible]
>MLOC_59921 -1041273
MQPDPSVGRPASVLPHKTANVRDHYRIGKKLGQGQFGTTYLCVAKEDGGEYACKSIPKRKLLCRED[illegible]
>MLOC_72770 -1041498
MGQCKPPGPVGEVLGRPIEDVRATYTIGEELGRGQFGVTYLCTHTETGEKLACKTIAKRKLSGAED[illegible]
>MLOC_6391 -1045437
M---SAGLLADSVLKRDTARLKDLYTIGKKLGQGQFGTTYLCVEKATGKEYACKSIAKRKLLTDED[illegible]
>MLOC_55774 -1045441
MGNTSAGLLVGSVLKRKTESVKEKYSLGRRLGQGQFGTTYLCVERSSGKEYACKSILKRKLVTDDD[illegible]
>MLOC_10811 -1045834
MGNCPPARAGLVVLRDP-RDLDDKYVLGGELGRGEFGVTYLCTEAATGARMACKSISKRKLRTPVD[illegible]
>MLOC_12765 -1048701
MGNRAGQVAMGRVLGRPMEDVRATYTFGRELGRGQFGVTYLVTHKATGQRFACKSIATRKLVHRDD[illegible]
>MLOC_54510 -1051450
MGNCKPPAAIGPVLGRPMEDVRSIYTVGKELGRGQFGVTSLCTHKATGQKFACKTIAKRKLSTKED[illegible]
>MLOC_43400 -1052287
[illegible]
>MLOC_7568 -1057507
CGGP-------------------------------ARTRTAPCTSSWSSARAASSS-------TASS[illegible]
>MLOC_37356 -1062636
[illegible]
>MLOC_79572 -1063201
MGQCPSKPTPDTILGKQYEDVRSVYSLGKELGRGQFGVTYLCTEISTGRQYACKSISKRKLVSKAD[illegible]
>MLOC_77271 -1063347
MGNTSAGLLAESVLKRDSERIKDLYTLGKKLGQGQFGTTYKCVEKATGKEFACKSIAKRKLVTEED[illegible]
>MLOC_71042 -1065939
[illegible]
>CHLREDRAFT_123538 -853939
------------------DVRDFYTFDKQLGKGNFGIVHLVFDKKTNEKYACKSISKRKLVTPED[illegible]
>CHLREDRAFT_59450 -855547
MGCSLGLEDGEVWITRQTTDIRKVFKFGQQINKGQFGVIHVVTDA-AGNKYACKQISKRKLTGPNS[illegible]
>CDPK1 -862211
MAKVPVVKPGNWILPTYDTDCWKDYEPGSILGKGTFGTTYSATNKKTGEKVAVKVISKKKLVSAEE[illegible]
>CHLREDRAFT_127871 -863336
MGDPRVSFSVANILGKGDPKLEDAYRVDKQIGKGAFGVVRAGAKRSTGDPVAVKSISKAKLVCKED[illegible]
>CHLREDRAFT_126628 -864848
MGCSPIDPEALWVFEETKELVKEHYAFGRILGKGQFGTCRTVTHKTTGKKYACKTIAKRRLFNMSD[illegible]
>A9TY96_PHYPA -870566
MGGFHSQPLGKRTNFGYERDFKEKYTLGKLLGHGQFGYTYVAIEKSTGNKVAAKCIEKKQMTLPIS[illegible]
>A9TXV1_PHYPA -870684
MGNCDRYYGNNQILKNQ-ARILDKYVLGRELGRGEFGITYLCTDKETQEVFACKSISKKKLRTAVD[illegible]
>A9TXV3_PHYPA -870687
MGNCDGYQTNNQILKNQ-ARILDKYVLGRELGRGEFGITYLCTDKETQEVFACKSISKKKLRTAVD[illegible]
>A9TUH0_PHYPA -872281
MGNTKMKSSSTGILGKPLRDIKLHYTLGRELGRGQFGVTYLCTDKETGISYACKTIAKRKLTNKDD[illegible]
>A7X9M2_9BRYO -872356
MGNQPVNVAAGTVLGKPLSDVRSVYTLGKELGRGQFGVTYACTNIKTGEHLACKSIAKRKLISKED[illegible]
>A9TSX5_PHYPA -872649
MAEV--QIHDFAVLQHKSEDVKDIYTLGKKLGEGQFGITYLCTEKATGLKYACKCIPKRKLISEEE[illegible]
>A9THK5_PHYPA -876751
M---DGYQTNLQILKNQ-SRILDKYNLGRELGRGEFGITYLCTDKETGEVFACKSISKKKLRTAVD[illegible]
>A7X9M1_9BRYO -877700
MGNTSGQSLTHSVLQRKTENLRDLYILGKKLGQGQFGTTYLCIEKATNKEYACKSIAKRKLISKED[illegible]
>A9T983_PHYPA -879391
----PGLSFTHSVLQRQTENLKDLYRLERKLGQGQFGTTYLCVEKATGREYACKSIAKRKLISQED[illegible]
>A7X9N1_9BRYO -879394
MGNTPGLSFTHSVLQRQTENLKDLYRLGRKLGQGQFGTTYLCVEKATGREYACKSIAKRKLISQED[illegible]
>A9T523_PHYPA -880991
MNPQKKGKLTVCVLQHVTEDVRDVYTLGKKLGAGNFGITYLCTEKSTGDDYACKTISKKKLKSKDD[illegible]
>A7X9N2_9BRYO -883009
MGNVPRSVSNVGVLGKPLVDIRQTYSLGKELGRGQFGVTYLCTHKETGEKLACKSIAKRKLIAKED[illegible]
>A9SYQ0_PHYPA -883423
MADF--HVPLPALLQNATDDLEKLYSLGKKLGEGQFGTTYLCTERATGLQFACKCIPKRKLISSEE[illegible]
>A9SRJ6_PHYPA -886275
MGNSKPKPVAPGFLGKPLSDILNSYTLGKELGRGEFGVTYTCTHKDTNEVYACKTIAKRKLTHKDD[illegible]
>A7X9L9_9BRYO -886281
MGNIPTSYSANGVLGKPLVDIRQTYSLGRELGRGQFGVTYLCTHKTTGEILACKSIAKRKLTTKED[illegible]
>A9SN80_PHYPA -887352
MGNCGDYHTNLQILKNQ-ARILDKYVLGRELGRGEFGITYLCTDKETQEIFACKSISKKKLRTAVD[illegible]
>E1C9R2_PHYPA -887676
MGNTPASRSVSGVLGKPLSDIRQSYILGRELGRGQFGVTYLCTDKMTNEAYACKSIAKRKLTSKED[illegible]
>A9SIW9_PHYPA -890073
MGGCHSQPLGKRTNFGYERDFKEKYSLGKLLGHGQFGYTYVATEKATGNKVAAKCIEKKQMKLPIS[illegible]
>A9S5F3_PHYPA -894944
M---PGQSFTHSVLQRNTENLKDLYTLGRKLGQGQFGTTYLCVEKTTGKEYACKSIAKRKLISQED[illegible]
>A9S5F3_PHYPA -894948
M---PGQSFTHSVLQRNTENLKDLYTLGRKLGQGQFGTTYLCVEKTTGKEYACKSIAKRKLISQED[illegible]
>A9RCS6_PHYPA -905109
MGNCDGFQSTNQILKNQ-ARILDKYVLGRELGRGEFGITYLCTDKETQDVFACKSISKKKLRTAVD[illegible]
>A9RCU5_PHYPA -905125
MGNCDGYQANSQILKNQ-AKILDKYILGRELGRGEFGITYLCTDKETGETLACKSISKKKLRTAVD[illegible]
>BRADI5G18250 -907723
MGNCKAGGAMGPVLQRPMVDVRSQYNLERKLGSGQFGTTYLCTERATGLKYACKSVSKRKLLRRAD[illegible]
>BRADI5G19430 -907870
MGNAAPADL-GSVLGHPTPSLRDLYQLGRKLGQGQFGTTFLCTELATGTEYACKSISKRKLITKED[illegible]
>BRADI4G07280 -909752
MGNVSAGLQVASVLRRKTENLKDKYSLGRKLGQGQFGTTYLCVDKASGQELACKSIAKRKLITDED[illegible]
>BRADI4G24390 -911417
MQTDASSGRPASVLPHKTANVRDHYRIGKKLGQGQFGTTYQCVAKEGGGDFACKSIPKRKLLCRED[illegible]
>BRADI4G26317 -911633
MGQCSSSAPVGEVLGRPMEDVRATYSIGKELGRGQFGVTHLCTHRTSGEKLACKTIAKRKLSTRED[illegible]
>BRADI4G35100 -912699
MGGCDGAGDSQAGPGS-CQCFSKRYRLGAELGRGEFGVTRRCVDAATGEALACKTIRRKRLRRGAD[illegible]
>BRADI4G40300 -913340
MQPDPSVGRPASVLPHKTANVRDHYRIGKKLGQGQFGTTYLCVAKEDGGEYACKSIPKRKLLCRED[illegible]
>BRADI4G43400 -913697
MGQCSSSAPVGEVLGNPIEDVRATYTIGEELGRGQFGVTYLCTHSITGEKLACKTIAKRKLSGKED[illegible]
>BRADI2G15520 -915728
MGARAAAEDTGRVLGRPLSDVRATYTFGRELGRGQFGVTYLATHKSTGARYACKSISARKLSRGDD[illegible]
>BRADI2G21390 -916450
MGNCKPPAPIGPVLGRPMEDVKSIYNVGKELGRGQFGVTSLCTHKATGHKFACKTISKRKLSTKED[illegible]
>BRADI2G22750 -916594
MGNCGGEKKLLSVLGEE-GGIDEKYALDRELGRGEFGVTYLCMDRGSKELLACKSISKRKLRTPVD[illegible]
>BRADI2G43910 -918681
MGNRAGVVAMGRVLGRPMEDVRATYTFGRELGRGQFGVTYLVTHKATGQRFACKSIATRKLVHRDD[illegible]
>BRADI2G52870 -919771
MGNCKPPAPIGPVLGRPMEDVRSIYTVGKELGRGQFGVTSLCTHKATGQKFACKTIAKRKLSTKED[illegible]
>BRADI2G54080 -919918
MGNCGGGQKRLAVLGED-GGIDDKYVLDRELGRGEFGVTYLCMDRDTKELLACKSISKRKLRTPVD[illegible]
>BRADI3G02600 -921205
MGGCGAEGVARVVEFGYERDFEGRYEVGRLLGHGQFGYTFAATDRGSGDRVAVKRIDKAKMNRPVA[illegible]
>BRADI3G32187 -924357
MGNS--SLRSTRVLERETPNIREHYTLGHKLGQGKFGTTYLCTEITTRCQYACKSILKAKFCHTED[illegible]
>BRADI3G41770 -925537
MGGCSPWEEEEPILRTTAAEFARRYVLGKELGRGEFGVTRRCKDAATGESLACKTIRRHRRRAAH[illegible]
>BRADI3G51970 -926795
MGNTAPAELTANVLGHPTPSLHDHYLLGRKLGQGQFGTTYLCTHRATGVDYACKSIGKRKLITKED[illegible]
>BRADI3G60750 -927874
MGNY----SPTAVLGHDTPPLTSLYTLGRKLGQGQFGTTYLCTDIATGTPLACKSIAKRKLLTPED[illegible]
>BRADI1G04440 -928427
MGNCTPARPGLVVLRDP-RDLEARYVLGGELGRGEFGITYLCTEAATGARLACKSISKRKLRTPVD[illegible]
>BRADI1G06270 -928657
MGNNSAGLQADSVLKRDTARLKDLYTIGKKLGQGQFGTTYLCVEKATGKEFACKSIAKRKLLTEED[illegible]
>BRADI1G06300 -928660
MGNTSAGLLVGSVLKRKTESVKEKYSLGRRLGQGQFGTTYLCVDRASGKEYACKSILKRKLVTDDD[illegible]
>BRADI1G12150 -929357
MGNAGP-----RVLDGVGPRVTDKYHLGRELGRGEFGVTRLATDRGSRERLACKSIPKARLRTAVD[illegible]
>BRADI1G24240 -930697
MGNCPPGATKLVVLREP-RDIAARYELGGELGRGEFGVTYLCTDRATGEALACKSISKKKLRTAVD[illegible]
>BRADI1G26310 -930946
MGQCPSKPTPDTILGKQYEDVRSVYSLGKELGRGQFGVTYLCTEIATGRQYACKSISKRKLVSKAD[illegible]
>BRADI1G52567 -933811
MGLCGIVACGKRTDFGYDKDFEARYTIGKLLGHGQFGYTFAAVDRYSDERVAVKRIDKNKMVLPVA[illegible]
>BRADI1G56970 -934301
MGNTSAGLSAESVLKRDSERIKDLYTLGKKLGQGQFGTTYQCVEKATGKEFACKSIAKRKLVTEED[illegible]
>BRADI1G76560 -936612
MGNQ--DPTSISVLGRKTADLRQNYILGRKLGQGQFGTTYLCTEISTGCEYACKTILKRKLITKVD[illegible]
>LOC_Os09g33910 -940670
MGGCGGGGRKNAILGDAAAGFAERYRLGAELGRGEFGVTRRCSDAATGEALACKTIRRKRLRCRGD[illegible]
>LOC_Os10g39420 -945060
MGNS--LNLKSPVLGYKTPNVRELYTLGRELGQGQFGKTYLCTEISTGCQYACKTILKSNLRCVSD[illegible]
>LOC_Os12g03970 -945915
MGQCKPAGPIGEVLERPMEEVRTTYSIGKELGRGQFGVTHLCTHKATGEKLACKTIAKRKLANKED[illegible]
>LOC_Os12g07230 -946303
MQPDPSVGRPASVLPHKTANVRDHYRIGKKLGQGQFGTTYLCVGKPDGGEYACKSIPKRKLLCRED[illegible]
>LOC_Os12g12860 -946910
MGNCM-SNEPATVLGNSAEEMLRRYEIGEELGRGEFGVTRRCRDAVTGERLACKSISKRKLRSSVD[illegible]
>LOC_Os12g30150 -948626
MGNVSAGLQVESVLRRKTENLKDKYSLGRKLGQGQFGTTYLCVDKANGGEYACKSIAKRKLLTDED[illegible]
>LOC_Os08g42750 -954969
MGGCDDGGDVVAIATTTADEFARRYVLGKELGRGEFGVTRRCSDAATGEALACKTIRKHRRLAAAH[illegible]
>LOC_Os11g04170 -955666
MGQCKPAGPIGEVLERPMEEVRTTYSIGKELGRGQFGVTHLCTHKATGEKLACKTIAKRKLANKED[illegible]
>LOC_Os11g07040 -956024
MQPDAAGGRPVSVLPHKTANVRDHYRIGKKLGQGQFGTTYLCVDKASGGEFACKSIPKRKLLCRED[illegible]
>LOC_Os07g06740 -960780
MGNTSAGLLADSVLKRDSERLKDLYTLGKKLGQGQFGTTYQCVEKATGKVLACKSIAKRKLVSEED[illegible]
>LOC_Os07g22710 -962362
MGLCGIVACGKRTDFGYDKDFEARYALGKLLGHGQFGYTFAAVDRRSSERVAVKRIDKNKMVLPVA[illegible]
>LOC_Os07g33110 -963510
MGSCPPKPTPDTILGKPYDDVRSVYSLGKELGRGQFGVTYLCTEIASGKQYACKSISKRKLVSKAD[illegible]
>LOC_Os07g38120 -964088
MGNCAPSGHRLVVLREP-RDIAARYELGGELGRGEFGVTYLCTERETGDAYACKSISKKKLRTAVD[illegible]
>LOC_Os05g39090 -969609
MGNCGWEKKRLSVLGEE-GGIEEKYALDRELGRGEFGVTYLCMDRCSRELLACKSISKRKLRTPVD[illegible]
>LOC_Os05g41270 -969877
MGNCKPPAPIGPVLGRPMEDVKSIYTVGKELGRGQFGVTSLCTHKATGQRFACKTISKRKLSTKED[illegible]
>LOC_Os05g50810 -970954
MGARAAEDGVGRVLGRPMEDVRATYTFGRELGRGQFGVTYLATHKPTGRRYACKSIAARKLARPDD[illegible]
>LOC_Os04g47300 -981655
MGNCASGGEMGPVLQRAMVSVRSLYQLDRKLGSGQFGTTYLCTERATGNRYACKSVSKRKLVRRTD[illegible]
>LOC_Os04g49510 -981919
MGNAAPADL-GSVLGHPTPNLRDLYAMGRKLGQGQFGTTYLCTELSTGVDYACKSISKRKLITKED[illegible]
>LOC_Os02g03410 -983489
MGACEKEKEAARVEFGYERDFEGRYQVGRLLGHGQFGYTFAATDRASGDRVAVKRIDKAKMVRPVA[illegible]
>LOC_Os02g46090 -988367
MGNTAPAELTANVLGHPTPSLSEHYALGRKLGQGQFGTTYLCTDLATGVDYACKSIAKRKLITKED[illegible]
>LOC_Os02g58520 -989996
MGNY----SPTAVLGHETPALREVYTVGRKLGQGQFGTTYLCTQVSTGAEYACKSIAKRKLLSPED[illegible]
>LOC_Os03g03660 -990411
MGNQ--DPTSINVLGRKTADLREHYIIGRKLGQGQFGTTYLCTEINTGCEYACKTIPKRKLITKED[illegible]
>LOC_Os03g48270 -995270
MGNTGAAASAVRVLEGV-LRVTDKYQLGRELGRGEFGVTHLATDRATRERLACKSIPKRRLRTAVD[illegible]
>LOC_Os03g57450 -996363
MGNTSAGLLVGSVLKRKTESLKDKYSLGRKLGQGQFGTTYLCVERATGKEFACKSILKRKLVTDDD[illegible]
>LOC_Os03g57510 -996370
MGNNSAGLLADSVLKRDTARLKDLYTIGKKLGQGQFGTTYLCVEKATGREFACKSIAKRKLLTQED[illegible]
>LOC_Os03g59390 -996588
MGNCAAGRPGLMVLRDP-RDLGARYELGGELGRGEFGITYLCTEAETGDRYACKSISKRKLRTPVD[illegible]
>AT1G35670 15219693
METKNPRRPSNTVLPYQTPRLRDHYLLGKKLGQGQFGTTYLCTEKSTSANYACKSIPKRKLVCRED[illegible]
>AT1G50700 15223629
MGNC--HSGAAAILEKPYEDVKLFYTLSKELGRGQFGVTYLCTEKSTGKRFACKSISKKKLVTKGD[illegible]
>AT2G38910 15224978
MGNTSAGLQIIDSVLGRKTENLKDIYSVGRKLGQGQFGTTFLCVDKKTGKEFACKTIAKRKLTTPED[illegible]
>AT2G31500 15225092
MGSC--FQPPSRVLPEPGDGIHLKYDLGKELGRGEFGVTHECIEISTRERFACKRISKEKLRTEID[illegible]
>AT2G17890 15227920
MGLCETIPYGKRVDFGYAKDFDHRYTIGKLLGHGQFGYTYVATDKKTGDRVAVKKIDKAKMTIPIA[illegible]
```

```
>AT3G10660 15228350
MGNASAGLRTESVLQRKTENFKEFYSLGRKLGQGQFGTTFLCLEKGTGNEYACKSISKRKLLTDEDVEDVRREIQIMHHLAGHPNVISIKGAYEDVVAVHLVMELCSGGELFDRIIQRGHYTERKAAELARTIVGVLEACHSLGVMHRDLKPENFLFVSREEDSLLKTIDFGLSMFFKPDEVFTDVVGSPYYVAPEVLRKRYGPESDVWSAGVIVYILLSGVPPFWAETEQGIFEQVLHGDLDFSSDPWPSISESAKDLVRKMLVRDPKRRLTAHQVLCHPWVQIDGVAPDKPLDSAVLSRMKQFSAMNKFKKMALRVIAESLSEEEIAGLKQMFKMIDADNSGQITFEELKAGLKRVGANLKESEILDLMQAADVDNSGTIDYKEFIAATLHLNKIEREDHLFAAFSYFDKDESGFITPDELQQACEEFGVEDARIEEMMRDVDQDKDGRIDYNEFVAMMQKGSIMGGPVGL
>AT3G20410 15231140
MGNCKTTTKSNSILENAFEDVKLFYTLGKELGRGQFGVTYLCTENSTGKKYACKSISKKKLVTKADKDDMRREIQIMQHLSGQPNIVEFKGAYEDEKAVNLVMELCAGGELFDRIIAKGHYTERAAASVCRQIVNVVKICHFMGVLHRDLKPENFLLSSKDEKALIKATDFGLSVFIEEGKVYRDIVGSAYYVAPEVLRRRYGKEVDIWSAGIILYILLSGVPPFWAETEKGIFDAILEGHIDFESQPWPSISSSAKDLVRRMLTADPKRRISAADVLQHPWLREGGEASDKPIDSAVLSRMKQFRAMNKLKKLALKVIAENIDTEEIQGLKAMFANIDTDNSGTITYEELKEGLAKLGSKLTEAEVKQLMDAADVDGNGSIDYIEFITATMHRHRLESNENLYKAFQHFDKDSSGYITIDELESALKEYGMGDATIKEVLSDVDSDNDGRINYEEFCAMMRSGNPQQQQR--
>AT4G09570 15233947
MEK-NPRRPSNSVLPYETPRLRDHYLLGKKLGQGQFGTTYLCTEKSSSANYACKSIPKRKLVCREDYEDVWREIQIMHHLSEHPNVVRIKGTYEDSVFVHIVMEVCEGGELFDRIVSKGCFSEREAAKLIKTILGVVEACHSLGVMHRDLKPENFLFDSPSDDAKLKATDFGLSVFYKPGQYLYDVVGSPYYVAPEVLKKCYGPEIDVWSAGVILYILLSGVPPFWAETESGIFRQILQGKIDFKSDPWPTISEGAKDLIYKMLDRSPKKRISAHEALCHPWIVDEHAAPDKPLDPAVLSRLKQFSQMNKIKKMALRVIAERLSEEEIGGLKELFKMIDTDNSGTITFEELKAGLKRVGSELMESEIKSLMDAADIDNSGTIDYGEFLAATLHINKMEREENLVVAFSYFDKDGSGYITIDELQQACTEFGLCDTPLDDMIKEIDLDNDGKIDFSEFTAMMKKGDGV-GRRTM
>AT4G04710 15234432
MGNCVSDQKQETILGKPLEDIKKHYSFGDELGKG--------------KSYACKSIPKRTLSSEEEKEAVKTEIQIMDHVSGQPNIVQIKGSYEDNNSIHIVMELCGGGELFDKIDAHSYYSEKDAAGIFRSIVNAVKICHSLDVVHRDLKPENFLFSSKDENAMLKAIDFGCSVYIKEGKTFERVVGSKYYIAPEVLEGSYGKEIDIWSAGVILYILLSGVPPFQTGIESIIVSEIKECRLDFESQPWPLISFKAKHLIGKMLTKKPKERISAADVLEHPWMK--SEAPDKPIDNVVLSRMKQFRAMNKLKKLALKVIAEGLSEEEIKGLKTMFENMDMDKSGSITYEELKMGLNRHGSKLSETEVKQLMEAADVDGNGTIDYIEFISATMHRHRLERDEHLYKAFQYFDKDGSGHITKEEVEIAMKEHGMGEANAKDLISEFDKNNDGKIDYEEFCTMMRNGILQ-PQKRL
>AT4G04720 15234435
MGCFVSVRDPDTILGKPFEDIRKFYSLGKELGRGQFGITYMCKEIGTGNTYACKSILKRKLISKQDKEDVKREIQIMQYLSGQPNIVEIKGAYEDRQSIHLVMELCAGGELFDRIIAQGHYSERAAAGIIRSIVNVVQICHFMGVVHRDLKPENFLLSSKEENAMLKATDFGLSVFIEEGKVYRDIVGSAYYVAPEVLRRSYGKEIDIWSAGVILYILLSGVPPFWAENEKGIFDEVIKGEIDFVSEPWPSISESAKDLVRKMLTKDPKRRITAAQVLEHPWIK-GGEAPDKPIDSAVLSRMKQFRAMNKLKKLALKVIAESLSEEEIKGLKTMFANIDTDKSGTITYEELKTGLTRLGSRLSETEVKQLMEAADVDGNGTIDYYEFISATMHRYKLDRDEHVYKAFQHFDKDNSGHITRDELESAMKEYGMGEASIKEVISEVDTDNDGRINFEEFCAMMRSGSTQ-PQKFH
>AT4G23650 15236560
MGHRTSTQQNGRILGRPMEEVRRTYEFGRELGRGQFGVTYLVTHKETKQQVACKSIPTRRLVHKDDIEDVRREVQIMHHLSGHRNIVDLKGAYEDRHSVNLIMELCEGGELFDRIISKGLYSERAAADLCRQMVMVVHSCHSMGVMHRDLKPENFLFLSKDENSPLKATDFGLSVFFKPGDKFKDLVGSAYYVAPEVLKRNYGPEADIWSAGVILYILLSGVPPFWGENETGIFDAILQGQLDFSADPWPALSDGAKDLVRKMLKYDPKDRLTAAEVLNHPWIREDGEASDKPLDNAVLSRMKQFRAMNKLKKMALKVIAENLSEEEIIGLKEMFKSLDTDNNGIVTLEELRTGLPKLGSKISEAEIRQLMEAADMDGDGSIDYLEFISATMHMNRIEREDHLYTAFQFFDNDNSGYITMEELELAMKKYNMGDKSIKEIIAEVDTDRDGKINYEEFVAMMKKGNPELVP-RR
>AT4G35310 15236966
MGNS-PDNQAYYVLGHKTPNIRDIYTLSRKLGQGQFGTTYLCTEIASGVDYACKSISKRKLISKEDVEDVRREIQIMHHLAGHGSIVTIKGAYEDSLYVHIVMELCAGGELFDRIIQRGHYSERKAAELTKIIVGVVEACHSLGVMHRDLKPENFLLVNKDDDFSLKAIDFGLSVFFKPGQIFTDVVGSPYYVAPEVLLKRYGPEADVWTAGVILYILLSGVPPFWAETQQGIFDAVLKGYIDFESDPWPVISDSAKDLIRRMLSSKPAERLTAHEVLRHPWICENGVAPDRALDPAVLSRLKQFSAMNKLKKMALKVIAESLSEEEIAGLREMFQAMDTDNSGAITFDELKAGLRKYGSTLKDTEIHDLMDAADVDNSGTIDYSEFIAATIHLNKLEREEHLVAAFQYFDKDGSGFITIDELQQACVEHGMADVFLEDIIKEVDQNNDGKIDYGEFVEMMQKGNAGVGRRTM
>AT5G23580 15237791
MAN---KPRTRWVLPYKTKNVEDNYFLGQVLGQGQFGTTFLCTHKQTGQKLACKSIPKRKLLCQEDYDDVLREIQIMHHLSEYPNVVRIESAYEDTKNVHLVMELCEGGELFDRIVKRGHYSEREAAKLIKTIVGVVEACHSLGVVHRDLKPENFLFSSSDEDASLKSTDFGLSVFCTPGEAFSELVGSAYYVAPEVLHKHYGPECDVWSAGVILYILLCGFPPFWAESEIGIFRKILQGKLEFEINPWPSISESAKDLIKKMLESNPKKRLTAHQVLCHPWIVDDKVAPDKPLDCAVVSRLKKFSAMNKLKKMALRVIAERLSEEEIGGLKELFKMIDTDKSGTITFEELKDSMRRVGSELMESEIQELLRAADVDESGTIDYGEFLAATIHLNKLEREENLVAAFSFFDKDASGYITIEELQQAWKEFGINDSNLDEMIKDIDQDNDGQIDYGEFVAMMRKGNGTIGRRTM
>AT5G04870 15238353
MGNTSAGLRTESVLQRKTENFKEFYSLGRKLGQGQFGTTFLCVEKTTGKEFACKSIAKRKLLTDEDVEDVRREIQIMHHLAGHPNVISIKGAYEDVVAVHLVMECCAGGELFDRIIQRGHYTERKAAELTRTIVGVVEACHSLGVMHRDLKPENFLFVSKHEDSLLKTIDFGLSMFFKPDDVFTDVVGSPYYVAPEVLRKRYGPEADVWSAGVIVYILLSGVPPFWAETEQGIFEQVLHGDLDFSSDPWPSISESAKDLVRKMLVRDPKKRLTAHQVLCHPWVQVDGVAPDKPLDSAVLSRMKQFSAMNKFKKMALRVIAESLSEEEIAGLKEMFNMIDADKSGQITFEELKAGLKRVGANLKESEILDLMQAADVDNSGTIDYKEFIAATLHLNKIEREDHLFAAFTYFDKDGSGYITPDELQQACEEFGVEDVRIEELMRDVDQDNDGRIDYNEFVAMMQKGSITGGPVGL
>AT5G19360 15239716
MGNCTKQGPIGPVLGRPMEDVKSSYTLGKELGRGQFGVTHLCTQKATGLQFACKTIAKRKLVNKEDIEDVRREVQIMHHLTGQPNIVELKGAYEDKHSVHLVMELCAGGELFDRIIAKGHYSERAAASLLRTIVQIIHTCHSMGVIHRDLKPENFLLLSKDENSPLKATDFGLSVFYKPGEVFKDIVGSAYYIAPEVLRRKYGPEADIWSIGVMLYILLCGVPPFWAESENGIFNAILSGQVDFSSDPWPVISPQAKDLVRKMLNSDPKQRLTAAQVLNHPWIKEDGEAPDVPLDNAVMSRLKQFKAMNNFKKVALRVIAGCLSEEEIMGLKEMFKGMDTDNSGTITLEELRQGLAKQGTRLSEYEVQQLMEAADADGNGTIDYGEFIAATMHINRLDREEHLYSAFQHFDKDNSGYITTEELEQALREFGMNGRDIKEIISEVDGDNDGRINYEEFVAMMRKGNPDPNP-KR
>AT5G19450 15239742
MGNCSGTGFKLSVLKDP-HDISLMYDLGREVGRGEFGITYLCTDIKTGEKYACKSISKKKLRTAVDIEDVRREVEIMKHMPRHPNIVSLKDAFEDDDAVHIVMELCEGGELFDRIVARGHYTERAAAAVMKTILEVVQICHKHGVMHRDLKPENFLFANKKETSALKAIDFGLSVFFKPGEGFNEIVGSPYYMAPEVLRRNYGPEVDIWSAGVILYILLCGVPPFWAETEQGVAQAIIRSVIDFKRDPWPRVSETAKDLVRKMLEPDPKKRLSAAQVLEHSWIQNAKKAPNVSLGETVKARLKQFSVMNKLKKRALRVIAEHLSVEEVAGIKEAFEMMDSKKTGKINLEELKFGLHKLGQQIPDTDLQILMEAADVDGDGTLNYGEFVAVSVHLKKMANDEHLHKAFSFFDQNQSDYIEIEELREALNDEVDTEEVVAAIMQDVDTDKDGRISYEEFAAMMKAGTDWRKARRE
>AT5G12180 15239888
MGNCTKQGPIGPVLGRPMEDVKASYSLGKELGRGQFGVTHLCTQKATGHQFACKTIAKRKLVNKEDIEDVRREVQIMHHLTGQPNIVELKGAYEDKHSVHLVMELCAGGELFDRIIAKGHYSERAAASLLRTIVQIVHTCHSMGVIHRDLKPENFLLLNKDENSPLKATDFGLSVFYKPGEVFKDIVGSAYYIAPEVLKRKYGPEADIWSIGVMLYILLCGVPPFWAESENGIFNAILRGHVDFSSDPWPSISPQAKDLVKKMLNSDPKQRLTAAQVLNHPWIKEDGEAPDVPLDNAVMSRLKQFKAMNNFKKVALRVIAGCLSEEEIMGLKEMFKGMDTDSSGTITLEELRQGLAKQGTRLSEYEVQQLMEAADADGNGTIDYGEFIAATMHINRLDREEHLYSAFQHFDKDNSGYITMEELEQALREFGMNGRDIKEIISEVDGDNDGRINYDEFVAMMRKGNPDPIP-KR
>AT5G12480 18416872
MGNCSGAGFKLSVLKDP-HDISLQYDLGREVGRGEFGITYLCTDKETGEKYACKSISKKKLRTAVDIEDVRREVEIMKHMPKHPNVVSLKDSFEDDDAVHIVMELCEGGELFDRIVARGHYTERAAAAVMKTIVEVVQICHKQGVMHRDLKPENFLFANKKETSALKAIDFGLSVFFKPGEQFNEIVGSPYYMAPEVLRRNYGPEIDVWSAGVILYILLCGVPPFWAETEQGVAQAIIRSVIDFKRDPWPRVSDSAKDLVRKMLEPDPKKRLTAAQVLEHTWILNAKKAPNVSLGETVKARLKQFSVMNKLKKRALRVIAEHLSVEEAAGIKEAFEMMDVNKRGKINLEELKYGLQKAGQQIADTDLQILMEATDVDGDGTLNYSEFVAVSVHLKKMANDEHLHKAFNFFDQNQSGYIEIDELREALNDELDTEEVIAAIMQDVDTDKDGRISYEEFVAMMKAGTDWRKARRE
>AT3G51850 22331739
MGNCGKKSAPIRVLSDV-ENIEDRYLLDRELGRGEFGVTYLCIERSSRDLLACKSISKRKLRTAVDIEDVKREVAIMKHLPKSSSIVTLKEACEDDNAVHLVMELCEGGELFDRIVARGHYTERAAAGVTKTIVEVVQLCHKHGVIHRDLKPENFLFANKKENSPLKAIDFGLSIFFKPGEKFSEIVGSPYYMAPEVLKRNYGPEIDIWSAGVILYILLCGVPPFWAESEQGVAQAILRGVIDFKREPWPNISETAKNLVRQMLEPDPKRRLTAKQVLEHPWIQNAKKAPNVPLGDVVKSRLKQFSVMNRFKRKALRVIAEFLSTEEVEDIKVMFNKMDTDNDGIVSIEELKAGLRDFSTQLAESEVQMLIEAVDTKGKGTLDYGEFVAVSLHLQKVANDEHLRKAFSYFDKDGNGYILPQELCDALKEDGGDVDVANDIFQEVDTDKDGRISYEEFAAMMKTGTDWRKARRG
>AT2G17290 30679935
MGNS-VDNQSYYVLGHKTPNIRDLYTLSRKLGQGQFGTTYLCTDIATGVDYACKSISKRKLISKEDVEDVRREIQIMHHLAGHKNIVTIKGAYEDPLYVHIVMELCAGGELFDRIIHRGHYSERKAAELTKIIVGVVEACHSLGVMHRDLKPENFLLVNKDDDFSLKAIDFGLSVFFKPGQIFKDVVGSPYYVAPEVLLKHYGPEADVWTAGVILYILLSGVPPFWAETQQGIFDAVLKGYIDFDTDPWPVISDSAKDLIRKMLCSSPSERLTAHEVLRHPWICENGVAPDRALDPAVLSRLKQFSAMNKLKKMALKVIAESLSEEEIAGLRAMFEAMDTDNSGAITFDELKAGLRRYGSTLKDTEIRDLMEAADVDNSGTIDYSEFIAATIHLNKLEREEHLVSAFQYFDKDGSGYITIDELQQSCIEHGMTDVFLEDIIKEVDQDNDGRIDYEEFVAMMQKGNAGVGRRTM
>AT3G57530 30694663
MGNCGGRPLKLIVLNDP-REIESKYTLGRELGRGEFGVTYLCTDKETDDVFACKSILKKKLRTAVDIEDVRREVEIMRHMPEHPNVVTLKETYEDEHAVHLVMELCEGGELFDRIVARGHYTERAAAAVTKTIMEVVQVCHKHGVMHRDLKPENFLFGNKKETAPLKAIDFGLSVFFKPGERFNEIVGSPYYMAPEVLKRNYGPEVDIWSAGVILYILLCGVPPFWAETEQGVAQAIIRSVLDFRRDPWPKVSENAKDLIRKMLDPDQKRRLTAQQVLDHPWLQNAKTAPNVSLGETVRARLKQFTVMNKLKKRALRVIAEHLSDEEASGIREGFQIMDTSQRGKINIDELKIGLQKLGHAIPQDDLQILMDAGDIDRDGYLDCDEFIAISVHLRKMGNDEHLKKAFAFFDQNNNGYIEIEELREALSDELG-EEVVDAIIRDVDTDKDGRISYEEFVTMMKTGTDWRKARRE
>AT1G74740 30699042
MGNCLRTHAPLRVIPMS-SQISDKYILGRELGRGEFGITYLCTDRETREALACKSISKRKLRTAVDVEDVRREVTIMSTLPEHPNVVKLKATYEDNENVHLVMELCEGGELFDRIVARGHYTERAAATVARTIAEVVRMCHVNGVMHRDLKPENFLFANKKENSALKAIDFGLSVLFKPGERFTEIVGSPYYMAPEVLKRNYGPEVDVWSAGVILYILLCGVPPFWAETEQGVALAILRGVLDFKRDPWSQISESAKSLVKQMLEPDSTKRLTAQQVLDHPWIQNAKKAPNVPLGDIVRSRLKQFSMMNRLKKKALRVIAEHLSIQEVEVIRNMFTLMDDDNDGKISYLELRAGLRKVGSQLGEPEIKLLMEVADVNGNGCLDYGEFVAVIIHLQKMENDEHFRQAFMFFDKDGSGYIESEELREALTDELGENSVIIDIMREVDTDKDGKINYDEFVVMMKAGTDWRKARRE
>Q14012 3122301
MLGA-----VEGPRWKQAEDIRDIYDFRDVLGTGAFSEVILAEDKRTQKLVAIKCIAKEALEGKE--GSMENEIAVLHKIK-HPNIVALDDIYESGGHLYLIMQLVSGGELFDRIVEKGFYTERDASRLIFQVLDAVKYLHDLGIVHRDLKPENLLYYSLDEDSKIMISDFGLSKMEDPGSVLSTACGTPGYVAPEVLQKPYSKAVDCWSIGVIAYILLCGYPPFYDENDAKLFEQILKAEYEFDSPYWDDISDSAKDFIRHLMEKDPEKRFTCEQALQHPWIAGD-TALDKNIHQSVSEQIKKNFAKSKWKQ-AFNATAV------VRHMRK-----------------LQLGTSQEG-QQTASHGELLTPVAGGPAAGCCCRDCCVEPGTELSPTLP---------------------HQL--------------------------------------------------
>NIASHv1004P07 326493696
------------------------------------------------------------------------------------------------------------------------------------------------------------------------------------------------------------------AGVILYILLCGVPPFWAETEQGVAQAILRGNIDFKREPWPHVSDNAKDLVRQMLQPDPKIRLTAKQVLEHTWLQNAKKAPNVPLGDIVKSRLKQFSRMNRFKRRALRVIADHLSAEEVEDIKEMFKVMDTDNDGIVSYEELKSGIAKFGSHLAESEVQMLIEAVDTNGRGALDYGEFLAVSLHLQRMANDEHLRRAFLFFDKDGDGFIEPEELQEALAEDGAVTEVVKDILQEVDTDKDGKISFEEFVAMMKTGTDWRKARRG
>MLOC_72357/NIASHv1012I21 326499562
MGACG----APAVEFGYDRDFDGRYEVGRLLGHGQFGYTFAAVERLSGDRVAVKRIDKAKMTRPVAVEDVKREVKILKALKGHENIVHFDNAFEDDSYVYIVMELCEGGELLDRILAKSRYSEKDAAVVVRQMLKVAAECHLRGLVHRDMKPENFLFKSTKEDSPLKATDFGLSDFIKPGKKFRDIVGSAYYVAPEVLKRRSGPESDVWSIGVITYILLCGRRPFWDKTEDGIFKEVLRNKPDFRKRPWPSISSSAKDFVKKLLVKNPRARLTAAQALSHPWVREGGDASEIPVDISVLYNMRQFVKYSRFKQFALRALASTVNEEELADLKDQFDAIDVDKSGSISIEEMRHALAKDLPRLKGPRVLEIIQAIDSNTDGLVDFKEFVAATLHIHQMAELDRCQAAFSKFDLDGDGYITPDELRMHTGL----KGSIEPLLEEADIDKDGRISLSEFRKLLRTASMS--NPGV
>MLOC_38029/NIASHv2129M13 326501394
MQPDAPSGRPASVLPHKTANVRDHYRIGKKLGQGQFGTTYLCVAKEDGGEFACKSIPKRKLLCREDYEDVWREIQIMHHLSEHPNVVRIRGAYEDALFVHIVMELCAGGELFDRIVAKGHYTERAAAQLIRTIVGVVEACHSLGVMHRDLKPENFLFASTAEDAPLKTTDFGLSMFYKPGDKFSDVVGSPYYVAPEVLQKCYGPEADVWSAGVILYILLCGVPPFWAETEAGIFRQILRGKLDFESEPWPSISDSAKDLVRTMLCRDPTKRLSAHEVLCHPWIVDDAVAPDKPIDSAVLSRLKHFSAMNKLKKMALRVIAESLSEEEIGGLRELFKMIDADNSGTITFDELKDGLKRVGSELTEHEIQALMDAADIDNSGTIDYGEFIAATLHMNKLEREENLVSAFSFFDKDGSGFITIDELSHACREFGLDDVHLEDMIKDVDQNNDGQIDYSEFTAMMRKGNAGTGRRTM
>NIASHv1023K05 326508913
MGLCGIVACGKRTDFGYDKDFEARYALGKLLGHGQFGYTFAAVNRHSDESVAVKRIDKNKMVLPVAVEDVKREVKILKALHGHENVVHFYNAFEDDNYVYIVMELCEGGELLDRILAKSRYSEKDAAVVVRQMLKVAAECHLHGLVHRDMKPENFLFKSSKEGSPLKATDFGLSDFITPGKQFHDIVGSAYYVAPEVLKRKSGPESDVWSIGVITYILLCGRRPFWDKTEDGIFKEVLKNKPDFRRKPWANITPSAKDFVQKLLVKDPHARLTAAQALSHEWVREGGQASEIPLDISVLHNMRQFVKYSRFKQFALRALASTLNPEELSDLRDQFNAIDIDKSGMISLEELKQALAKDVPRLKGPRVLEIVEAIDSNTDGFVDFEEFVAATLHMHQLVEHDLSQAAFDKFDVDGDGYITSNELRMNTGL----KGSIDPLLEEADIDKDGKISLDEFRKLLKTASMRSCN-SV
>NIASHv3144K21 326527871
MGGCGGGDDDQCVPGAGGECFAKRYRLGAELGRGEFGVTRRCEDAATGEALACKTIRRKRLRRAADAEDVRREVEILRRMSAEGTVVRLREACEDAEGVHLVMELCEGGELFDRIFARGHYTERAAAKIGRTIARVVQLCHDNGVMHRDLKPENFLFAGKEEDSPLKAIDFGLSVYFQPGERFTEVVGSGIYMAPEVLMRSYGPEADVWSAGVILYILLCGVPPFWGDTDERIAESIIRGEINFEREPWPKVSHTAKDLVKKMLDPDPATRLTANQVSEHPWLKNADKAPNVSLGELVRSRLKQFSSMNKFKKKALGVVAKSLPAEEIENYTQMFQTMDKDKDGTLTLEELKEGLRINGHPVPESEIQMLLEAGDIDGNGTLDTEEFVTVLLHIKKKSNEEYLPEAFKYFDKDGNGYIEMEELMEALGDDELGEQVIKDIIRDVDTDEDGRISYQEFEVMMRSGSDWRNARRA
>AT4G04740 334186354
MGCFISVRDPETILGKPFEDIRKFYSLGRELGRGGLGITYMCKEIGTGNIYACKSILKRKLISELGREDVKTEIQIMQHLSGQPNVVEIKGSYEDRHSVHLVMELCAGGELFDRIIAQGHYSERAAAGTIKSIVDVVQICHLNGVIHRDLKPENFLFSSKEENAMLKVTDFGLSAFIEEGKIYKDVVGSPYYVAPEVLRQSYGKEIDIWSAGVILYILLCGVPPFWADNEEGVFVEILKCKIDFVREPWPSISDSAKDLVEKMLTEDPKRRITAAQVLEHPWIK-GGEAPEKPIDSTVLSRMKQFRAMNKLKKLALKVSAVSLSEEEIKGLKTLFANMDTNRSGTITYEQLQTGLSRLRSRLSETEVQQLVEASDVDGNGTIDYYEFISATMHRYKLHHDEHVHKAFQHLDKDKNGHITRDELESAMKEYGMGEASIKEVISEVDTDN-----ALSPVEIMREVALEVQEGPV
>AT4G21940 334186798
MGCFIVFRETETILGKPFEEIRKLYTLGKELGRGQFGITYTCKENSTGNTYACKSILKRKLTRKQDIDDVKREIQIMQYLSGQENIVEIKGAYEDRQSIHLVMELCGGSELFDRIIAQGHYSEKAAAGVIRSVLNVVQICHFMGVIHRDLKPENFLLASTDENAMLKATDFGLSVFIEEGKVYRDIVGSAYYVAPEVLRRSYGKEIDIWSAGIILYILLCGVPPFWSETEKGIFNEIIKGEIDFDSQPWPSISESAKDLVRKLLTKDPKQRISAAQALEHPWIR-GGEAPDKPIDSAVLSRMKQFRAMNKLKKLALKVIAESLSEEEIKGLKTMFANMDTDKSGTITYEELKNGLAKLGSKLTEAEVKQLMEAADVDGNGTIDYIEFISATMHRYRFDRDEHVFKAFQYFDKDNSGFITMDELESAMKEYGMGEASIKEVIAEVDTDNDGRINYEEFCAMMRSGITLPQQKPC
>AT4G36070 334187210
MGLCGGIPYGKRIDFGYAKDFDNRYTIGKLLGHGQFGFTYVATDNNNGNRVAVKRIDKAKMTQPIEVEDVKREVKILQALGGHENVVGFHNAFEDKTYIYIVMELCDGGELLDRILAKSRYTEKDAAVVVRQMLKVAAECHLRGLVHRDMKPENFLFKSTEEGSSLKATDFGLSDFIKPGVKFQDIVGSAYYVAPEVLKRRSGPESDVWSIGVITYILLCGRRPFWDKTQDGIFNEVMRKKPDFREVPWPTISNGAKDFVKKLLVKEPRARLTAAQALSHSWVKEGGEASEVPIDISVLNNMRQFVKFSRLKQIALRALAKTINEDELDDLRDQFDAIDIDKNGSISLEEMRQALAKDVPKLKDARVAEILQANDSNTDGLVDFTEFVVAALHVNQLEEHDRSRAAFDKFDIDGDGFITPEELRLQTGL----KGSIEPLLEEADVDEDGRISINEFRRLLRSASLKSKNKGT
>AT4G38230 334187258
MGLA-GGNQACYVLGQKTPSIRDLYSLGHKLGQGQFGTTYMCKEISTGREYACKSITKRKLISKEDVEDVRREIQIMHHLAGYKNIVTIKGAYEDPLYVHIVMELCSGGELFDRIIQRGHYSERKAAELIKIIVGVVEACHSLGVMHRDLKPENFLLVNKDDDFSLKAIDFGLSVFFKPGQIFEDVVGSPYYVAPEVLLKHYGPEADVWTAGVILYILVSGVPPFWAETQQGIFDAVLKGHIDFDSDPWPLISDSAKNLIRGMLCSRPSERLTAHQVLRHPWICENGVAPDRALDPAVLSRLKQFSAMNKLKQMALRVIAESLSEEEIAGLKEMFKAMDTDNSGAITFDELKAGLRRYGSTLKDTEIRDLMEAADIDKSGTIDYGEFIAATIHLNKLEREEHLLSAFRYFDKDGSGYITIDELQHACAEQGMSDVFLEDVIKEVDQDNDGRIDYGEFVAMMQKGIVG---RTM
>AT1G18890 42562158
MGNCTRSPAPIRVLKDV-TQISDKYILGRELGRGEFGITYLCTDRETHEALACKSISKRKLRTAVDIEDVRREVAIMSTLPEHPNVVKLKASYEDNENVHLVMELCEGGELFDRIVARGHYTERAAAAVARTIAEVVMMCHSNGVMHRDLKPENFLFANKKENSPLKAIDFGLSVFFKPGDKFTEIVGSPYYMAPEVLKRDYGPGVDVWSAGVIIYILLCGVPPFWAETEQGVALAILRGVLDFKRDPWPQISESAKSLVKQMLDPDPTKRLTAQQVLAHPWIQNAKKAPNVPLGDIVRSRLKQFSMMNRFKKKVLRVIAEHLSIQEVEVIKNMFSLMDDDKDGKITYPELKAGLQKVGSQLGEPEIKMLMEVADVDGNGFLDYGEFVAVIIHLQKIENDELFKLAFMFFDKDGSTYIELDELREALADELGEASVLSDIMREVDTDKDGRINYDEFVTMMKAGTDWRKARRE
>AT1G61950 42562873
MGCLSTLQQPEPILGRPFEDIKEKYSLGRELGRGQFGITYICTEISSGKNFACKSILKRKLIRTKDREDVRREIQIMHYLSGQPNIVEIKGAYEDRQSVHLVMELCEGGELFDKITKRGHYSEKAAAEIIRSVVKVVQICHFMGVIHRDLKPENFLLSSKDEASSLKATDFGVSVFIEEGKVYEDIVGSAYYVAPEVLKRNYGKAIDIWSAGVILYILLCGNPPFWAETDKGIFEEILRGEIDFESEPWPSISESAKDLVRNMLKYDPKKRFTAAQVLEHPWIREGGEASDKPIDSAVLSRMKQLRAMNKLKKLAFKFIAQNLKEEELKGLKTMFANMDTDKSGTITYDELKSGLEKLGSRLTETEVKQLLEDADVDGNGTIDYIEFISATMNRFRVEREDNLFKAFQHFDKDNSGFISRQELETAMKEYNMGDIMIKEIISEVDADNDGSINYQEFCNMMKSCSQS-HQKPN
>AT4G04700 42566304
MGCFELQQSKRTILEKPLVDITKIYILGEELGRGNFGLTRKCVEKSTGKTFACKTILKTKLKDEECEEDVKREIRIMKQLSGEPNIVEFKNAYEDKDSVHIVMEYCGGGELYDKILAGKSYSEKEAAGIIRSIVNVVKNCHYMGVMHRDLKPENFLLTSNDDNATVKVIDFGCSVFIEEGKVYQDLAGSDYYIAPEVLQGNYGKEADIWSAGIILYILLCGKSPFVKEPEGQMFNEIKSLEIDYSEEPWPLRDSRAIHLVKRMLDRNPKERISAAEVLGHPWMKE-GEASDKPIDGVVLSRLKRFRDANKFKKVVLKFIAANLSEEEIKGLKTLFTNIDTDKSGNITLEELKTGLTRLGSNLSKTEVEQLMEAADMDGNGTIDIDEFISATMHRYKLDRDEHVYKAFQHFDKDNDGHITKEELEMAMKEDGAGEGSIKQIIADADTDNDGKINFEEFRTMMRTESSLQPEEII
>AT4G04695 42570056
MGCYNLKQSKRTILEKPFVDIGKVYILGDELGQGQFGITRKCVEKTSGKTYACKTILKTNLKSREDEEAVKREIRIMKHLSGEPNIVEFKKAYEDRDSVHIVMEYCGGGELFKKIEAGKSYSEKEAVEIIRPIVNVVKNCHYMGVMLRDLKPENFLLSSTDKNATVKAIDFGCSVFIEEGEVHRKFAGSAYYIAPEVLQGKYGKEADIWSAGIILYILLCGKPPFVTEPEAQMFSEIKSAKIDVDSESWKFIDVKAKHLVNRMLNRNPKERISAAEVLGHPWMKD-GEASDKPIDGVVLSRLKQFRDMNKLKKVALKVIAANLSEEEIKGLKTLFTNIDTDKSGTITLEELKTGLTRLGSNLSKTEVEQLMEAADVDGNGTIDIDEFISATMHRYRLDRDDHVYQAFQHFDKDNDGHITKEELEMAMKEHGVGEVSIKQIITEVDTDNDGKINFEEFRTMMRSGSSLQPQEIK
>AT2G41860 42571175
MGNCHHDGLKLIVLKEP-HEIKQKYKLGRELGRGEFGVTYLCTEIETGEIFACKSILKKKLKTSIDIEDVKREVEIMRQMPEHPNIVTLKETYEDDKAVHLVMELCEGGELFDRIVARGHYTERAAASVIKTIIEVVQMCHKHGVMHRDLKPENFLFANKKETASLKAIDFGLSVFFKPGDRFNEIVGSPYYMAPEVLRRSYGQEIDIWSAGVILYILLCGVPPFWAETEHGVAKAILKSVIDFKRDPWPKVSDNAKDLIKKMLHPDPRRRLTAQQVLDHPWIQNGKNASNVSLGETVRARLKQFSVMNKLKKRALRVIAEHLSVEETSCIKERFQVMDTSNRGKITITELGIGLQKLGIVVPQDDIQILMDAGDVDKDGYLDVNEFVAISVHIRKLGNDEHLKKAFTFFDKNKSGYIEIEELRDALADDVDTEEVVEAIILDVDTNKDGKISYDEFATMMKTGTDWRKARRD
>AT1G76040 79607733
MGFCSSGSQIGPILNRPMIDLSALYDLHKELGRGQFGITYKCTDKSNGREYACKSISKRKLIRRKDIEDVRREVMILQHLTGQPNIVEFRGAYEDKDNLHLVMELCSGGELFDRIIKKGSYSEKEAANIFRQIVNVVHVCHFMGVVHRDLKPENFLLVSNEEDSPIKATDFGLSVFIEEGKVYRDIVGSAYYVAPEVLHRNYGKEIDVWSAGVMLYILLSGVPPFWGETEKTIFEAILEGKLDLETSPWPTISESAKDLIRKMLIRDPKKRITAAEALEHPWMTD-TKISDKPINSAVLVRMKQFRAMNKLKKLALKVIAENLSEEEIKGLKQTFKNMDTDESGTITFDELRNGLHRLGSKLTESEIKQLMEAADVDKSGTIDYIEFVTATMHRHRLEKEENLIEAFKYFDKDRSGFITRDELKHSMTEYGMGDATIDEVINDVDTDNDGRINYEEFVAMMRKGTTD-SDK--
>AK249631 __AK249361.1
------------------------------------------------------------------------------------------------------------------------------------------MCHSLGVMHRDLKPENFLLLDKDDDLSIKAIDFGLSVYFKPGQVFSELVGSPFYVAPEVLHKRYGPESDVWSAGVILYVLLSGVPPFWADTQKGIFDAVLKGHLDLESDPWPKISDSAKDLIRKMLCNCPSERLKAHEVLRHPWICENGPATDGVLDPSVISRLKRFSAMNNLQKLALRVIAERLSEEEIAGLRELFKTVDIKNSGVITFGELRKGLTRYGNGLVDTEICDIMEAADTDTDVTINYEEFIAATMPLNKIEREEHLKAAFTYFDKDGSGYITVDKLQLACAEYNMEGTLLEEIISEADQNNDGQIDYAEFVAMMQGNANGLGR-TM
>MLOC_58648 __MLOC_58648
----SAGLQVESVLRRKTDNLKDKYSLGRKLGQGQFGTTYLCVDKATALEYACKSIAKRKLITDEDVEDVRREIQIMHHLAGHPNIISIRGAYEDAVAVHVVMELCAGGELFDRIVRRGHYTERQAAELARVIAVVESCHSLGVMHRDLKPENFLFVGNEEDAALKTIDFGLSMFFRPGEIFSDVVGSPYYVAPEVLKKNYSQEADVWSAGVIVYILLCGVPPF--------------------------------------------------------------------------------------------------------------------------------------------------------------------------------------------------------------------------------------------------------
>MLOC_71733 __MLOC_71733
------------------------------------------------------------------------------------------------------------------------------------------------------------------------------------------------MAPEVLRRSYGPEVDIWSAGVILYILLCGVPPFWAETEQGVARSILRGALDLDREPWPRISDSAKSLVRQMLHMDPRKRLTARQVLAHPWLQDAKKAPNVPLGDVVRARLKQFSVMNRFKKKAMRVIAEHLSAEEVEVIKEMFAFMDTDNNGRVTLEELKAGLARVGSKLAEPEMELLMEAADVDGDGYLDYAEFVAITIHLQRLSNDQHLRKAFLFFDRDSSGYIERAELADALIDDSGKDAVVDHVLQEVDTDKDGRVSFEEFVAMMKAGTDWRKARRQ
```
